# Balancing the Oral Redox State: Endogenous and Exogenous Sources of Reactive Oxygen Species and the Antioxidant Role of Lamiaceae and Asteraceae

**DOI:** 10.3390/dj13050222

**Published:** 2025-05-21

**Authors:** Caterina Nela Dumitru, Lupoae Mariana, Cristian Constantin Budacu, Gabriela Mitea, Marius Daniel Radu, Alina Oana Dumitru, Andreea Lupoae, Alin Tatu, Gabi Topor

**Affiliations:** 1 Department of Pharmaceutical Sciences, Faculty of Medicine and Pharmacy, “Dunărea de Jos” University, 800010 Galati, Romania; caterina.dumitru@ugal.ro (C.N.D.); gabi.topor@ugal.ro (G.T.); 2 Department of Dentoalveolar and Maxillofacial Surgery, Faculty of Dental Medicine, “Grigore T. Popa” University of Medicine and Pharmacy, 700115 Iasi, Romania; 3 Department of Pharmacology, Faculty of Pharmacy, Ovidius University of Constanța, 900470 Constanța, Romania; gabriela.mitea@365.univ-ovidius.ro; 4 Faculty of Natural and Agricultural Sciences, Ovidius University of Constanța, 900470 Constanța, Romania; marius.radu@univ-ovidius.ro; 5 Faculty of Medicine and Pharmacy, “Dunărea de Jos” University, 800010 Galati, Romania; ad539@student.ugal.ro; 6 Emergency Clinical Hospital “St. Apostle Andrew”, 800010 Galati, Romania; al186@student.ugal.ro; 7 Clinical Medical Department, Faculty of Medicine and Pharmacy, “Dunărea de Jos” University, 800008 Galati, Romania; alin.tatu@ugal.ro

**Keywords:** oxidative stress, oral health, medicinal plants, antioxidant potential, reactive oxygen species, antioxidant activity, oral diseases, polyphenols, phytotherapy, Lamiaceae and Asteraceae families

## Abstract

**Background/Objectives:** Oral health is a complex concept involving physical, psychological, emotional, and social components. A key factor in maintaining oral tissue integrity is redox balance, which is disrupted by oxidative stress (OS) through an imbalance between reactive oxygen species (ROS) and antioxidant defenses. This study examines the contribution of endogenous and exogenous sources to OS and explores the therapeutic potential of medicinal plants from the Asteraceae and Lamiaceae families in restoring redox homeostasis and improving oral health. **Methods:** A literature review was conducted, analyzing the role of OS in oral diseases and the antioxidant mechanisms of selected Asteraceae species. Special attention was given to their phytochemical contents—polyphenols, flavonoids, and essential oils—and their biological relevance to oral health. **Results:** OS plays a critical role in the onset and progression of oral conditions such as caries, periodontitis, gingivitis, aphthous ulcers, abscesses, precancerous lesions, and oral cancers. ROS and reactive nitrogen species (RNS) cause inflammation, tissue breakdown, and salivary gland dysfunction. Asteraceae plants like *Matricaria chamomilla*, *Calendula officinalis*, *Cichorium intybus*, *Taraxacum officinale*, *Arctium lappa*, *Achillea millefolium*, and *Solidago virgaurea* demonstrate notable antioxidant, anti-inflammatory, and antimicrobial properties that help counteract OS and support oral homeostasis. **Conclusions:** Asteraceae and Lamiaceae species show high therapeutic potential in addressing OS-related oral disorders. Their bioactive compounds aid in restoring redox balance and protecting oral tissues. These findings support the integration of phytotherapeutic agents into oral healthcare and call for further clinical validation of plant-based strategies for disease prevention and management.

## 1. Introduction

Oral health is a complex and multidimensional concept, integrating physical, psychological, emotional, and social aspects that are essential for overall well-being [1]. Maintaining redox balance plays a crucial role in preventing the deterioration of oral structures, with OS being a key determinant in this dynamic.

The concept of OS has been defined as an imbalance between ROS and antioxidant systems, thereby disrupting redox signaling and regulation, leading to molecular damage [2]. Free radicals, such as ROS and RNS, have a dual role: they are necessary for physiological mechanisms but become harmful in excess, affecting fundamental biological structures.

ROS include radicals such as superoxide anions (O_2_^−^), hydroxyl radicals (•OH), alkoxyl (RO•), and peroxyl (ROO•), as well as non-radical forms like hydrogen peroxide (H_2_O_2_) and singlet oxygen (^1^O_2_). RNS comprise nitric oxide (NO), nitrogen dioxide (NO_2_), and peroxynitrite (ONOO^−^) [3]. These reactive species are produced endogenously, through cellular metabolism, and exogenously, from sources such as UV radiation, pollution, smoking, and diets rich in transition metals [4].

The main endogenous sources of ROS are mitochondria, where electron leakage generates O_2_^−^. At the same time, mitochondria generate superoxide radicals (O_2_•^−^) at multiple sites, including complex I and III, glycerol 3-phosphate dehydrogenase, and other dehydrogenases, with release into both the mitochondrial matrix and the intermembrane space. These ROS are further converted by mitochondrial enzymes, such as Mn-SOD and Cu/Zn-SOD, and may contribute to OS in inflammation and pathological conditions like ischemia/reperfusion [5].

ROS have emerged as critical secondary messengers in cellular signaling pathways regulating proliferation, differentiation, apoptosis, and inflammatory responses [3]. However, sustained ROS overproduction—particularly superoxide anions generated under chronic hyperglycemic conditions—has been strongly implicated in the onset and progression of both type 1 and type 2 diabetes mellitus, as well as in related complications such as cardiovascular dysfunction, neurodegeneration (including Alzheimer’s and Parkinson’s diseases), atherosclerosis, diabetic retinopathy, and nephropathy [6]. In oral inflammatory diseases, ROS exacerbate tissue destruction by enhancing local inflammation, directly damaging periodontal cells, and activating proteolytic enzymes that degrade the extracellular matrix [7].

RNS, such as NO and ONOO^−^, are produced by nitric oxide synthase (NOS). NO has protective roles in vasodilation, neuronal signaling, and immune response, but under chronic inflammatory conditions it reacts with superoxide (O_2_^−^) to form ONOO^−^, a potent oxidative agent that damages lipids, proteins, and DNA. The interaction between ROS and RNS intensifies OS, activating pro-inflammatory pathways such as NF-κB and increasing the production of inflammatory cytokines. Additionally, ONOO^−^ contributes to atherosclerosis, hypertension, and oral tissue degradation, accelerating periodontitis and precancerous lesions [8].

To counteract OS, the body utilizes enzymatic antioxidants (superoxide dismutase—SOD, catalase—CAT, glutathione (GSH), peroxidase—GPx) and non-enzymatic antioxidant molecules (vitamins C and E, coenzyme Q10, polyphenols, flavonoids, and essential minerals such as zinc and selenium) [9,10]. Saliva plays a central role in oral protection through the activity of its antioxidant enzymes and the presence of bioactive compounds [11].

Interest in using medicinal plants to prevent and treat oral diseases has significantly increased due to their antioxidant potential. One in three modern pharmaceutical drugs originates from plants, emphasizing the importance of natural resources in therapy [12]. Throughout history, medicinal plants have been a fundamental component of traditional oral care, due to their ability to provide bioactive compounds with proven therapeutic roles—including antimicrobial, anti-inflammatory, and antioxidant effects—which contribute to maintaining oral health and preventing various dental conditions [13].

**Figure 1:**
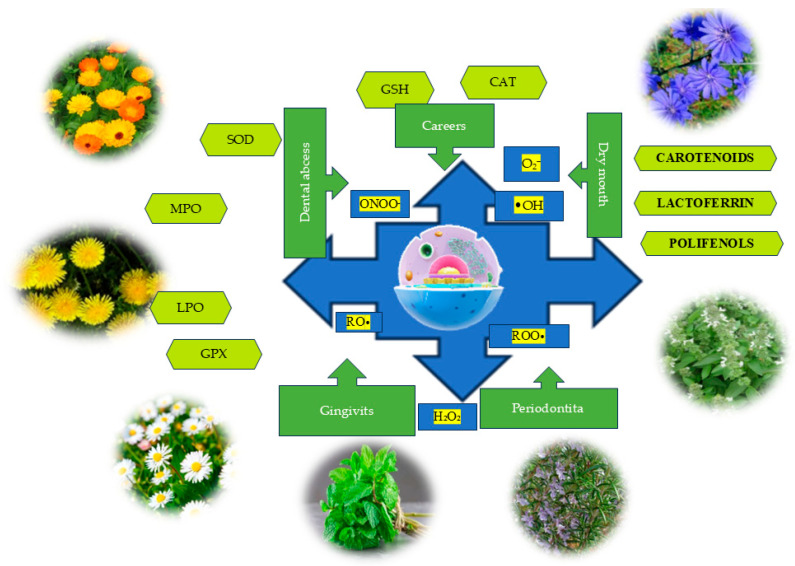
The benefits of plants in addressing oral disorders related to oxidative stress.

The present study explores the endogenous and exogenous sources of ROS involved in oral OS, along with their impact on salivary homeostasis. It also evaluates the antioxidant potential of plants from the Lamiaceae and Asteraceae families in protecting oral tissues, contributing to the maintenance of redox balance and the prevention of OS-induced degradation (Figure 1).

## 2. Materials and Methods

This article is a narrative review of the scientific literature, aiming to synthesize and interpret existing data on the role of OS in oral pathology, as well as to explore the therapeutic potential of medicinal plants, particularly those belonging to the Asteraceae and Lamiaceae families. The search strategy involved consulting major international scientific databases, including PubMed, Scopus, Web of Science, and Google Scholar. Only articles published after the year 2000 were considered eligible for inclusion, in order to ensure the relevance and contemporaneity of the data.

For the presentation and discussion of the results, the extracted information was thematically organized, with particular emphasis on the identification of both endogenous and exogenous sources of ROS, as well as on the antioxidant mechanisms of action of bioactive compounds from the selected plant families. The analysis focused on the contribution of these ROS sources to redox imbalance, as well as the therapeutic relevance of phytochemicals in the prevention and management of OS-related oral diseases.

The search was conducted using the following keywords: oxidative stress, oral health, medicinal plants, antioxidant potential, reactive oxygen species, antioxidant activity, oral diseases, polyphenols, phytotherapy, and Lamiaceae and Asteraceae families. Articles written in English and Romanian were included, with priority given to peer-reviewed narrative reviews, original experimental and clinical studies, systematic reviews, and meta-analyses.

Studies were selected based on their relevance to the topic, methodological rigor, and scientific contribution. Publications lacking clear data on phytochemical composition or antioxidant activity, as well as non-peer-reviewed sources, were excluded from the analysis.

## 3. Sources of Oxidative Stress

**Figure 2:**
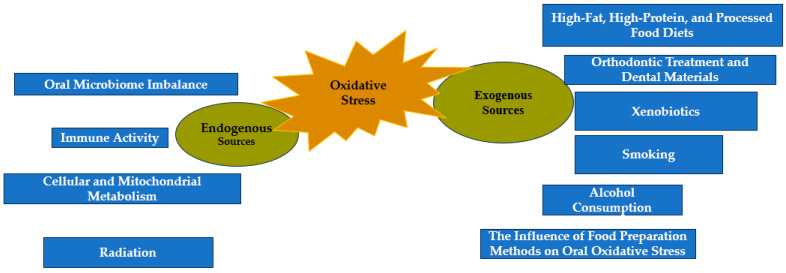
Endogenous and exogenous sources of reactive oxygen species.

Figure 2 illustrates the main endogenous and exogenous sources contributing to oral OS. These sources interact in complex ways to disrupt redox balance and contribute to tissue damage and inflammation in the oral cavity.

### 3.1. Endogenous Sources

#### 3.1.1. Cellular and Mitochondrial Metabolism

Mitochondria are essential for cellular energy production through oxidative phosphorylation and the regulation of redox homeostasis, playing significant roles in immunity, apoptosis, and cell growth [14,15].

Under pathological conditions, pathogens, senescence, and toxin exposure can lead to mitochondrial dysfunction, characterized by respiratory chain impairment, ATP depletion, and excessive mtROS accumulation. This results in a vicious cycle of OS and cellular damage. It can also lead to the release of damage-associated molecular patterns (DAMPs), which activate immune recognition receptors, triggering inflammation via neutrophil activation, NLRP3 inflammasome stimulation, and cytokine production. Additionally, it influences macrophage, T-cell, and B-cell metabolism, contributing to inflammation and autoimmune diseases [7].

Studies have shown that *Porphyromonas gingivalis* (*P. gingivalis*), a pathogenic bacterium associated with periodontitis progression, promotes lipid accumulation in macrophages, a key process in atherosclerotic plaque formation [16]. Furthermore, *P. gingivalis* infection induces mitochondrial fragmentation, disrupting redox balance and reducing ATP production in vascular endothelial cells. Research suggests that this effect is linked to the translocation of dynamin-related protein 1 (Drp1), a crucial factor in mitochondrial fission. This fragmentation contributes to mitochondrial dysfunction and vascular injury, exacerbating inflammation and the progression of cardiovascular diseases associated with periodontitis [17].

Dental caries represents a primary etiological factor in the onset and progression of pulpitis. Recent studies have demonstrated that irreversible pulpitis is associated with dysregulated mitochondrial dynamics, characterized by upregulated expression of dynamin-related protein 1 (Drp1), a key mediator of mitochondrial fission, alongside a significant downregulation of mitochondrial fusion proteins such as mitofusin 2 (MFN2) and optic atrophy 1 (OPA1) [18]. These alterations suggest enhanced mitochondrial fragmentation and functional impairment. Moreover, pulpal inflammation secondary to carious lesions has been strongly correlated with disruptions in the OS response within the dental pulp microenvironment. This imbalance is evidenced by the excessive accumulation of ROS, which contribute to cellular damage and the amplification of the inflammatory cascade [18]. As shown by Dogan Buzoglu et al. [19], increased ROS production in inflamed pulp tissue is accompanied by elevated levels of reduced GSH, indicating the activation of endogenous antioxidant defenses aimed at counteracting oxidative injury. The elevated OS observed in both pulpitis and periodontitis has been further associated with exacerbated dental pain and alveolar bone degradation, underscoring its central role in the pathophysiology of oral inflammatory diseases [20].

Mitochondrial dysfunction caused by the deficiency of SOD2 in salivary gland ductal cells induces local OS that leads to reversible glandular hypofunction, demonstrating that mitochondrial OS directly contributes to the development of hyposalivation, even in the absence of the autoimmune inflammation typical of Sjögren’s syndrome [21].

In Sjögren’s syndrome, mitochondrial dysfunction plays a role in pathogenesis, with structural mitochondrial alterations observed in salivary gland cells, disrupted energy metabolism, and changes in mitochondrial dynamics-related genes. Increased levels of mitochondrial double-stranded RNA (mt-dsRNA) and lactate in the salivary glands have been linked to immune system activation and exacerbated inflammation [22,23,24].

#### 3.1.2. Immune Activity

Immune activity represents the coordinated set of responses orchestrated by the host’s defense system to identify, neutralize, and eliminate pathogenic threats. It encompasses both innate and adaptive mechanisms, involving cellular components such as neutrophils, macrophages, and lymphocytes, as well as molecular mediators like cytokines and ROS [25]. In the oral cavity, immune activity is continuously engaged due to constant exposure to microbial biofilms, necessitating a delicate balance between protective inflammation and tissue homeostasis.

ROS are generated both by activated phagocytes—such as granulocytes, monocytes, and macrophages, as a defense mechanism against pathogenic microorganisms—and by oral bacteria. A key process in this context is the respiratory burst, where phagocytes rapidly increase their oxygen consumption, leading to O_2_^−^ production. This is converted into H_2_O_2_, an essential compound for pathogen elimination. While this mechanism is crucial for microbial destruction, ROS can also damage host tissues by oxidizing proteins, lipids, and cellular DNA [26]. ONOO^−^, formed through the rapid reaction between NO and superoxide radicals during phagocytic activation, is a potent oxidant that mediates antimicrobial defense but also contributes to local OS by damaging proteins, lipids, and mitochondrial function [27].

Polymorphonuclear leukocytes, including neutrophils, serve as the first line of cellular defense against bacteria in the gingival sulcus. They use both oxygen-dependent and oxygen-independent mechanisms to combat infections. The oxygen-dependent mechanism involves ROS production, which destroys bacteria but may also contribute to periodontal tissue degradation by damaging DNA, triggering lipid peroxidation, and oxidizing proteins and enzymes [28].

Unlike circulating neutrophils, oral neutrophils produce superoxide and NO even without external stimulation. This sustained activity can contribute to OS, affecting oral health. An increase in gingival crevicular fluid, rich in inflammatory markers and ROS, is observed in chronic inflammation, such as gingivitis and periodontitis. Mixing with saliva can intensify OS on adjacent oral structures [27].

OS plays a central role in the progression of periodontal diseases by affecting collagen structure, stimulating osteoclast activity, and promoting bone resorption. These effects highlight the need for a balance between immune defense mechanisms and host tissue protection to maintain oral health. Maintaining this balance is essential for preventing tissue degradation associated with inflammatory oral diseases.

#### 3.1.3. Oral Microbiome Imbalance

The imbalance of the oral microbiome, known as dysbiosis, refers to a disruption in the equilibrium between commensal and pathogenic microorganisms in the oral cavity. This imbalance can be triggered by factors such as poor oral hygiene, a sugar-rich diet, smoking, stress, or antibiotic use, which promote the selection of pro-inflammatory bacteria and contribute to the development of inflammatory oral diseases such as dental caries and periodontitis [29]. Under dysbiotic conditions, pathogenic bacteria can induce OS, amplifying local inflammation and compromising the integrity of oral tissues.

Beyond phagocyte activity, certain oral bacteria directly contribute to ROS production as an adaptation mechanism to the oral environment. A notable example is *Streptococcus mutans* (*S. mutans*), a facultative anaerobic bacterium involved in dental caries formation. It can generate O_2_^−^ and H_2_O_2_ through an NADH-oxidase-based enzymatic system. This enzyme catalyzes molecular oxygen reduction, resulting in H_2_O_2_ formation and the elimination of residual peroxides. Thus, although *S. mutans* lacks CAT or peroxidase, it can survive in aerobic environments similarly to strictly aerobic bacteria [30].

### 3.2. Exogenous Sources

#### 3.2.1. Alcohol Consumption

Alcohol consumption significantly increases ethanol, acetaldehyde, and ROS concentrations in saliva compared to serum. Within approximately 30 min, salivary ethanol concentration stabilizes and becomes similar to serum levels. In contrast, acetaldehyde and ROS persist longer, leading to their accumulation in the oral mucosa [31].

A key factor in this process is the enzyme ADH, which is present in the oral mucosa but with much lower activity than in the liver. As a result, the conversion of acetaldehyde to acetate is slowed, leading to the accumulation of acetaldehyde—a compound with high toxic potential [32].

Free radicals are generated throughout all stages of alcohol metabolism. Ethanol’s conversion to acetaldehyde involves ADH, the microsomal ethanol-oxidizing system (MEOS), and CAT. Among these, ADH and MEOS are the primary sources of free radicals such as O_2_^−^ and OH•. Subsequently, acetaldehyde’s oxidation to acetate by aldehyde dehydrogenase (ALDH) further generates ROS, amplifying OS [33].

Another major mechanism involves the acetaldehyde-induced activation of cytochrome P450 enzymes (CYP2E1, CYP1A2, and CYP3A4) in the oral cavity, which enhances the formation of free radicals, particularly superoxide and H_2_O_2_ [34]. These compounds damage proteins, lipids, and cellular DNA by forming adducts with amino acids and sulfhydryl groups, contributing to alcohol-induced toxicity in the oral mucosa and salivary glands [35].

Chronic alcohol consumption profoundly affects salivary gland function, particularly the parotid glands, which are more sensitive to OS than the submandibular glands. Studies indicate that alcohol reduces unstimulated salivary flow, impairs protein secretion, and decreases salivary amylase activity. These changes are associated with chronic inflammation, oral mucosal ulcerations, precancerous lesions, and, in the long term, an increased risk of oral and pharyngeal cancers due to ROS-induced oxidative modifications [34].

Not only chronic alcohol consumption but also a single episode of excessive alcohol intake can rapidly increase OS. During acute ethanol intoxication, mitochondrial superoxide production intensifies, and the activation of phospholipases A1 and A2 leads to the release of arachidonic acid, further amplifying ROS generation. Additionally, a decrease in peroxidase activity has been observed, suggesting an increased consumption of this enzyme for free radical neutralization [35].

These findings indicate that alcohol profoundly disrupts the antioxidant systems of the oral cavity, promoting oxidative lesions and salivary dysfunctions. Repeated ethanol exposure contributes to chronic inflammation, tissue structure alteration, and, in the long term, the development of degenerative oral diseases.

#### 3.2.2. Smoking

Cigarette smoke contains over 4800 toxic substances, more than 400 of which have pro-oxidative and carcinogenic effects, impacting both oral and systemic health. Heavy metals like cadmium interfere with the metabolism of zinc, iron, and copper, disrupting essential cellular functions. By inhibiting iron’s transport and utilization, cadmium promotes free iron accumulation in cells, where it participates in the Fenton reaction, catalyzing ROS production. This process, alongside quinone, semiquinone, and hydroquinone radicals in cigarette smoke, leads to additional H_2_O_2_, superoxide (O_2_^−^), and OH• formation, intensifying OS and cellular damage [36,37].

Toxic compounds in cigarette smoke contribute to carcinogenesis by modifying DNA bases, increasing the risk of genetic mutations. Smoking promotes DNA strand breakage, affecting genomic stability, inactivating tumor-suppressor genes, and activating proto-oncogenes. These molecular processes play a crucial role in malignant cell transformation, explaining the increased incidence of oral cancer among smokers. Studies show that smoking accelerates oxidative DNA damage, favoring mutagenic processes and the development of oral neoplasms [38].

Beyond the direct effects of toxic compounds, the interaction among tobacco use, OS–antioxidant imbalance, and genetic susceptibility synergistically contributes to the initiation of carcinogenesis in predisposed individuals [39].

Smokers are 2.6 to 6 times more likely to develop periodontal disease compared to non-smokers and show a reduced response to periodontal treatment. Additionally, the success rate of dental implants is lower among smokers, highlighting the negative impact of smoking on oral health [40].

#### 3.2.3. Xenobiotics

Xenobiotics are foreign substances to the body, present in food, drugs, and pollutants, undergoing electron transfer oxidation processes that generate ROS. These compounds can enter reduction–oxidation cycles, disrupting cellular metabolism and redox balance. Due to their unpredictable and potentially severe impacts, there is significant public concern regarding environmental contamination by pharmaceutical residues [41].

Among the most important xenobiotics with pro-oxidative effects are heavy metals, pesticides, industrial pollutants, and endocrine disruptors. Heavy metals such as arsenic, cadmium, mercury, lead, nickel, and chromium contribute to ROS generation either through direct redox reactions or by disrupting antioxidant systems. These metals can interfere with cellular functions, creating a redox imbalance that damages biological structures [42].

In addition to metals, pesticides and herbicides, such as organophosphates and paraquat, can induce severe OS. Their metabolism generates ROS, leading to cellular component damage and metabolic dysfunctions. Prolonged exposure to these substances is associated with chronic inflammation and tissue dysfunction [43].

Another harmful category of xenobiotics includes industrial pollutants such as polycyclic aromatic hydrocarbons (PAHs), polychlorinated biphenyls (PCBs), dioxins, and furans. These compounds form reactive metabolites that significantly increase OS, promoting tissue damage and impairing normal cellular function [44].

Endocrine disruptors, such as bisphenol A (BPA) and phthalates, including di(2-ethylhexyl) phthalate (DEHP), also influence redox homeostasis and trigger oxidative responses. These compounds can disrupt hormonal balance and cellular metabolism, amplifying the risk of OS and tissue damage [45].

Iron and copper play a crucial role in catalyzing Fenton and Haber–Weiss reactions, in which H_2_O_2_ interacts with these metals, generating highly reactive hydroxyl radicals [46]. An imbalance in metal ions, through either exposure to xenobiotic metals or internal dysfunctions, amplifies ROS and RNS generation, disrupting cellular redox signaling [38]. Copper exposure, exacerbated by industrial activities such as mining and fertilizer use, contributes to environmental contamination. Studies indicate that this exposure increases the risk of dental caries, leukoplakia, submucosal fibrosis, and oral cancer, while high copper concentrations in carious teeth suggest a direct role of OS in dental deterioration [46,47,48].

Lead substitutes calcium in hydroxyapatite (HA), reducing dental crystal size and compromising enamel integrity, which increases the incidence of caries and enamel hypoplasia. This association underscores the importance of monitoring lead levels and reducing environmental contamination [45,49,50].

Cadmium, a toxic metal, binds to thiol (-SH) groups in mitochondrial membrane proteins, disrupting permeability and essential ion transport. This interference affects the respiratory chain, with complex III inhibition leading to semiquinone accumulation, which transfers electrons to oxygen, generating O_2_^−^ and amplifying ROS production [51,52].

Arsenic, a toxic metalloid, enters the body through contaminated water, food, and polluted air. Chronic exposure is associated with gingival diseases, delayed healing of oral lesions, and an increased risk of oral cancer. Its toxicity stems from its ability to disrupt cell division and DNA repair, and to induce OS, leading to its accumulation in bones and teeth, affecting their structural integrity [53,54].

Mercury, a heavy metal with toxic effects on the body, originates from food, dental amalgam fillings, and industrial emissions. In dentistry, amalgam fillings contain approximately 50% mercury and can release vapors during chewing, abrasion, or bruxism [55]. These vapors are absorbed into the bloodstream and can be stored in the bones and teeth, causing systemic effects, including oral inflammation, metallic taste, and allergic reactions [55,56].

Under normal conditions, O_2_^−^ and H_2_O_2_ are relatively harmless. However, in the presence of redox-active metal ions, these compounds are converted into highly reactive radicals, amplifying OS. In the Fenton reaction, metal ions catalyze the conversion of H_2_O_2_ into HO•, the most reactive oxidative radical, while the Haber–Weiss reaction contributes to the regeneration of metal ions through their reduction by O_2_^−^, intensifying the production of hazardous radicals [57].

These reactions highlight the importance of controlling metal ion concentrations in the body, as even low levels can trigger a chain effect, amplifying oxidative cellular damage. Therefore, implementing effective antioxidant defense mechanisms, such as monitoring toxic metal levels and reducing exposure to harmful xenobiotics, is essential [58].

#### 3.2.4. Orthodontic Treatment and Dental Materials

Dental materials, including resin composites, cements, root canal filling materials, and dental alloys, can affect the balance between the formation and neutralization of free radicals, thereby contributing to OS [59]. The application of laser treatment to both soft and hard dental tissues significantly increases the production of free radicals in the oral cavity. Additionally, photodynamic laser therapy stimulates ROS formation, exerting a bactericidal effect, but also having pro-oxidative potential [60].

Nickel–chromium and cobalt–chromium-based alloys are frequently used in prosthetic restorations due to their mechanical strength and low cost. However, the corrosion of these materials can lead to the release of metal ions into the oral cavity, which may be absorbed by the body through the gastrointestinal tract. The released ions can cause allergic reactions, local and systemic toxicity, carcinogenic changes, and alterations in cellular structure and function [61].

Nickel and cobalt ions can interact with H_2_O_2_, generating •OH through Fenton and Haber–Weiss reactions. Moreover, chromium and cobalt ions can initiate redox cycles that directly contribute to free radical production. These mechanisms amplify OS and may have a significant impact on both oral and systemic health [59,61].

Studies have shown that metallic dental restorations can contribute to increased OS in the oral cavity. Numerous studies have demonstrated that dental metallic restorations, particularly amalgam fillings, can significantly contribute to OS in the oral cavity [62]. The electrochemical environment of the mouth facilitates galvanic reactions between the metals in amalgam and saliva, leading to the continuous release of mercury and other metal ions into oral tissues. Mercury, in particular, promotes the generation of ROS, alters the redox potential of oral cells, and causes sustained oxidative damage to soft tissues. Clinically, elevated salivary levels of malondialdehyde (MDA)—a key lipid peroxidation marker—have been positively correlated with the number of amalgam-filled surfaces [63]. Furthermore, chronic exposure to mercury appears to deplete key antioxidant systems, including total salivary GSH, which is vital both for detoxifying heavy metals and for counteracting oxidative insults. Studies show elevated levels of the OS marker MDA in the saliva of patients with amalgam fillings compared to those with composite or glass ionomer cement restorations, suggesting that amalgam may act as a chronic source of local OS, with potential impacts on the oral mucosa and overall redox balance [64]. Tomova found that unstimulated saliva in patients with such restorations exhibited high levels of 8-iso-prostaglandin F_2_-alpha (8-isoPGF_2_-α), a marker of lipid peroxidation, indicating local redox imbalance [59]. This effect was less pronounced in stimulated saliva, suggesting that both the presence of metallic materials and the composition of the alloys influence OS levels. However, the study was limited by a small patient sample and a lack of details on the specific types of alloys used [59].

These findings highlight the importance of evaluating the biocompatibility of dental materials to minimize OS and its long-term effects on oral health.

#### 3.2.5. High-Fat, High-Protein, and Processed Food Diets

Calorie-rich diets containing high amounts of proteins, carbohydrates, and fats contribute to increased OS and inflammation at the molecular and cellular levels. Excessive fat accumulation stimulates mitochondrial β-oxidation, leading to additional ROS generation. This, combined with the activation of the NF-κB transcription factor and excessive NO production, triggers pro-inflammatory signaling and promotes the accumulation of RNS [65]. The resulting OS leads to cellular dysfunction, apoptosis, cytotoxicity, and DNA mutations, contributing to molecular and tissue damage and the progression of chronic diseases.

A high-fat diet increases free fatty acid levels through chylomicrons, which are absorbed by the liver and directed toward β-oxidation or triglyceride esterification. Their excess promotes the generation of low-density lipoprotein (LDL) and oxidized LDL, contributing to macrophages’ transformation into foam cells and the formation of atherosclerotic plaques associated with cardiovascular diseases [65].

Animal model studies have shown that high-fat diets increase ROS production and reduce the antioxidant capacity of the parotid and submandibular glands. This imbalance affects the synthesis and secretion of salivary proteins, negatively influencing salivary flow. Stimulated secretion of salivary glands is reduced, which may compromise the lubrication and protection of the oral cavity [66].

A high-protein diet promotes increased free radical production in the oral cavity, leading to DNA, protein, and lipid oxidation in the salivary glands. The parotid gland, with a predominantly aerobic metabolism, is more resistant to OS, whereas the submandibular gland, with lower oxygen consumption, is more vulnerable to ROS accumulation. Excess protein intake can reduce the total salivary protein content and decrease stimulated salivary flow, impairing saliva’s defensive capacity against pathogens, its digestive function, and its role in protecting the oral mucosa [67]. Although some studies suggest that the parotid gland is more sensitive to OS—particularly in the context of chronic alcohol consumption—due to its rich vascularization and metabolic activity, other evidence indicates that its predominantly aerobic metabolism may actually confer greater resistance to oxidative damage when compared to the submandibular gland. These seemingly contradictory observations may stem from differences in experimental design, type of oxidative insult (e.g., alcohol vs. protein overload), and glandular response under stimulated vs. unstimulated conditions. Therefore, the relative susceptibility of the salivary glands to OS may vary depending on the specific oxidative trigger, metabolic demands, and adaptive antioxidant responses. Further comparative studies are needed to clarify these context-dependent vulnerabilities.

Excessive consumption of fermented foods, such as cheese, may cause temporary microbiota changes and is associated with a transient increase in OS. Other studies suggest a positive correlation between simple carbohydrate intake and the total antioxidant capacity of saliva [68], possibly due to increased UA levels in the blood [69].

#### 3.2.6. The Influence of Food Preparation Methods on Oral OS

Food preparation methods can significantly impact OS levels in the oral cavity by generating reactive compounds and exposing oral tissues to high temperatures. High-temperature cooking methods, such as frying and intensive thermal processing, promote the formation of reactive aldehydes, such as acrolein. This α,β-unsaturated compound exhibits high reactivity with nucleophiles, particularly thiol groups, leading to a reduction in reduced GSH, the primary cellular antioxidant. Studies have shown that acrolein inhibits thioredoxin and GSH activity, diminishing cellular defense mechanisms against OS [70].

Thermal stimuli have been shown to enhance oxidative stress in dental pulp tissues by promoting ROS production, as demonstrated in both healthy and inflamed pulp models [19]. Among these, nuclear factor Nrf2 plays a central role by translocating into the nucleus and stimulating the expression of antioxidant enzymes such as heme oxygenase-1 (HO-1). Simultaneously, Nrf2 activation can induce the expression of inflammatory genes, including interleukin-8 (IL-8), which may contribute to exacerbating oral inflammation [71].

These findings suggest that high-temperature food preparation and frequent consumption of hot foods may increase OS in the oral cavity, affecting redox homeostasis and amplifying local inflammation. Future studies should explore the impact of these factors on the oral microbiome and salivary antioxidant balance.

#### 3.2.7. Radiation

Ionizing radiation used in the treatment of head and neck cancers is a well-documented exogenous source of OS in the oral cavity. Radiotherapy, while effective against malignant cells, inevitably affects the surrounding healthy tissues, including the oral mucosa, salivary glands, and alveolar bone [72]. The resulting increase in ROS production disrupts redox homeostasis, impairs microvascular circulation, and promotes chronic inflammation. In particular, mandibular osteoradionecrosis (ORN), a severe late adverse effect of RT, is characterized by progressive bone necrosis, pain, and infection, frequently following dental extractions or mucosal injury [73]. Studies have shown that irradiated oral tissues experience sustained oxidative damage, with elevated levels of 8-oxo-7,8-dihydro-2’-deoxyguanosine (8-oxo-dG) serving as a biomarker of DNA oxidation. This persistent OS contributes to extracellular matrix degradation, delays healing, and increases susceptibility to secondary infections. Genetic susceptibility, such as polymorphisms in the *GSTP1* gene involved in ROS detoxification, further modulates the individual risk of radiation-induced oral complications [72]. Thus, radiotherapy represents a critical exogenous factor that is capable of initiating and perpetuating OS-related pathology within the oral environment.

## 4. Mechanisms by Which Free Radicals Induce OS in the Oral Cavity

### 4.1. Lipid Peroxidation and Cellular Membrane Destruction

ROS generated in the oral cavity can initiate lipid peroxidation by attacking polyunsaturated fatty acids (PUFAs) in cellular membranes. Hydroxyl radicals formed, for example, through the Fenton reaction catalyzed by labile iron, can abstract hydrogen from the bis-allylic groups of PUFAs, generating carbon-centered lipid radicals stabilized by resonance. These lipid radicals rapidly react with molecular oxygen, forming peroxyl radicals (ROO•), which, in turn, can attack other nearby unsaturated fatty acids, propagating an autocatalytic chain reaction [74].

In the propagation phase, lipid hydroperoxides and new radicals accumulate, altering the physical properties of the cellular membrane and causing covalent modifications to neighboring proteins and DNA. Radical lipid peroxidation typically evolves in three phases—initiation, propagation, and termination. Initiation involves the generation of radicals from non-reactive molecules, often mediated by transition metals (primarily iron) through Fenton chemistry [75]. In this process, ferrous ion (Fe^2+^) converts H_2_O_2_ into the highly reactive •OH, which triggers lipid attack. During propagation, the formed peroxyl radicals extend the peroxidative cascade, with each attack generating new radicals and lipid peroxides. Termination occurs when two radicals combine (neutralizing each other) or when antioxidants intervene, donating electrons to radicals without themselves becoming radicals, thus breaking the chain reaction. Oxidoreductase enzymes such as lipoxygenases (LOX), cyclooxygenases (COX), and cytochrome P450 (CYP) can also generate lipid peroxides in a controlled manner, exacerbating OS when their products accumulate pathologically [75].

MDA is a major secondary product of PUFA peroxidation and an important indicator of oxidative lipid membrane destruction [76]. The degradation of hydroperoxides formed in the peroxidative chain results in reactive fragments such as MDA, making MDA determination a conventional marker of OS.

Clinical evidence suggests the involvement of lipid peroxidation in inflammatory and neoplastic lesions of the oral cavity. In chronic periodontal disease, excessive local OS has been observed, with activated neutrophils producing large amounts of ROS that can damage periodontal tissues. Tsai reported significantly elevated MDA levels in the gingival crevicular fluid and gingival tissue of periodontitis patients compared to healthy controls [77], suggesting that lipid peroxidation exacerbates periodontal destruction. Smoking—an external factor that intensifies oral OS—exacerbates these effects. In his study, Kurtul [78] showed that smokers had significantly higher salivary MDA levels than non-smokers, proportional to the number of cigarettes consumed. This indicates that smoking increases lipid peroxidation in the oral cavity, contributing to oxidative damage to the mucosal tissues. An imbalance in membrane peroxidation has also been documented in oral carcinogenesis. Rai B. [79] reported increased MDA levels in both precancerous oral lesions and patients with oral carcinoma compared to healthy subjects. Gokul et al. [80] confirmed that patients with OSSC exhibited an altered oxidant–antioxidant status, with an accumulation of lipid peroxidation products in tumors and circulation, along with a decrease in endogenous antioxidant defense.

These findings suggest that ROS-mediated lipid peroxidation contributes both to periodontal tissue destruction and to the initiation and progression of oral neoplasms, particularly in the presence of risk factors such as chronic inflammation and smoking.

### 4.2. Oxidation of Salivary and Enzymatic Proteins

Proteins and enzymes in the oral cavity play structural, catalytic, and regulatory roles in maintaining local homeostasis. OS affects their integrity, leading to enzyme inactivation and metabolic pathway disruption. Unlike nucleic acids, oxidized proteins cannot be repaired and must be eliminated through proteolysis to prevent their accumulation and subsequent destructive effects on cells. ROS can damage proteins through amino acid residue oxidation, peptide bond cleavage, and protein aggregation [81].

Protein carbonyl groups (PCs) are stable products of protein oxidation and serve as biomarkers of OS. Serum and salivary PC levels are correlated with the severity of periodontitis, being elevated in patients with advanced periodontal disease [82]. Free radicals can induce the formation of carbon-centered protein radicals (P•), which undergo further oxidation, dimerization, or proteolytic degradation [83].

Protein oxidation involves modifications to both the main chain and amino acid side chains, resulting in the formation of protein peroxyl radicals (POO•). γ-Radiolysis studies have demonstrated that amino acids such as valine, leucine, and lysine generate peroxyl radicals, contributing to irreversible protein damage [84]. In the case of tyrosine, reaction with hydroxyl radicals produces phenoxyl radicals, which, in the presence of oxygen, lead to the formation of 3,4-dihydroxyphenylalanine. Similarly, the oxidation of tryptophan residues generates N-formylkynurenine and kynurenine. Cysteine, an essential amino acid in salivary protein structures, is particularly susceptible to oxidation. Thiol radicals (RS•), formed through the reaction of thiol compounds with ROS, can interact with oxygen, generating thiol peroxyl radicals. These radicals subsequently transform into sulfenic, sulfinic, or sulfonic acids, affecting proteins’ biological activity. Post-translational modifications through S-nitrosylation and S-glutathionylation can alter enzyme function via interactions with RNS or GSH [85]. Protein peroxyl radicals (POO•) can initiate chain reactions leading to fragmentation, dimerization, or irreversible protein alterations [84]. Proteins exposed to prolonged OS undergo more severe modifications than those affected by radiation or Fenton-type reactions. For example, lysozyme loses half of its activity after the oxidation of 42 amino acid residues following γ-radiation exposure, whereas the Fenton reaction requires the modification of only 13.5 residues to produce the same effect [86].

Oxidation of salivary proteins plays a crucial role in oral OS, affecting their structure and function, and influencing oral health and various systemic pathologies. Protein peroxyl radicals and chain oxidative reactions contribute to the alteration of salivary enzymes and proteins, disrupting oral homeostasis.

### 4.3. Nucleic Acid Degradation and DNA Mutations

Nucleic acid degradation and DNA mutations are major consequences of OS induced by ROS. These reactive species directly interact with DNA, causing damage to purine and pyrimidine bases, and affecting genetic stability. A frequently used biomarker for quantifying such damage is 8-hydroxy-deoxyguanosine (8-OHdG), which indicates oxidative DNA deterioration. Studies have reported elevated levels of 8-OHdG in gingival crevicular fluid (GCF) and saliva in periodontitis patients, significantly correlating with disease severity [87].

DNA oxidation not only compromises genome stability but also affects other biomolecules, leading to the propagation of oxidative damage. Peroxidized proteins can react with DNA, forming covalent intermolecular bonds that impact enzymatic functionality and genetic structure [88]. The proposed mechanism involves the initial formation of RO• radicals through a metal-mediated reaction bound to DNA, followed by their interaction with DNA molecules, resulting in cross-linking between oxidized proteins and the genetic structure.

Any protein oxidized by ROS can form such bonds with DNA, either through diffusion or during normal functional interactions. These DNA–protein modifications have drawn attention due to their implications in oxidative lesion induction and repair mechanisms, as well as genomic integrity [89,90]. In the oral cavity, such alterations may contribute to enzymatic dysfunction, modifications in salivary protein properties, and increased susceptibility to inflammatory and degenerative diseases.

### 4.4. Activation of the Inflammatory Response and Pro-Oxidant Enzymes

The activation of the inflammatory response and pro-oxidant enzymes by ROS plays a central role in the pathogenesis of periodontal disease. ROS are produced both physiologically and in response to periodontal pathogens, contributing to inflammation and tissue degradation. In chronic inflammation, ROS overproduction exacerbates tissue destruction by oxidizing essential biomolecules and activating signaling pathways involved in inflammation and cell death [91].

Studies indicate increased levels of ROS metabolites in the sera of patients with chronic periodontitis, suggesting a heightened systemic OS burden [92]. A major mechanism through which ROS contribute to tissue damage is the activation of gingival fibroblasts, which, in response to bacterial infections, produce ROS and amplify local inflammation [93].

Exposure of periodontal tissues to high ROS concentrations leads to leukocyte infiltration and connective tissue degradation, impacting the extracellular matrix by destroying proteoglycans, hyaluronan, and collagen, thereby weakening the structural support of the periodontium [94].

The impact of ROS extends to bone metabolism, inhibiting osteoblast differentiation and stimulating osteoclastogenesis, which favors bone resorption characteristic of periodontal disease [95]. In vitro studies have demonstrated that hydroxyl radicals and H_2_O_2_ degrade alveolar bone proteoglycans, accelerating their destruction [95].

Another crucial mechanism through which OS exacerbates periodontal disease is the activation of matrix metalloproteinases (MMPs), enzymes responsible for extracellular matrix degradation. ROS activate MMP-2, MMP-8, MMP-9, and MMP-13, which promote periodontal tissue destruction. Furthermore, MMPs modulate cytokine and growth factor bioactivity, amplifying the inflammatory response [96]. ROS can also stimulate the NF-κB pathway, which regulates the expression of pro-inflammatory cytokines (IL-1β, TNF-α, IL-6) and matrix metalloproteinases, intensifying tissue degradation [97]. Additionally, ROS activate the JNK (c-Jun N-terminal kinase) pathway, leading to cellular apoptosis and periodontal tissue breakdown [98]. Studies have shown that JNK inhibitors reduce pro-inflammatory cytokine production, suggesting a crucial role for this mechanism in periodontitis [99].

Another key element in periodontal disease progression is the activation of the NLRP3 inflammasome, which is elevated in the tissues of periodontitis patients and activates caspase-1, promoting the release of IL-1β and IL-18, and amplifying inflammation. Recent evidence suggests that ROS activate the NLRP3 inflammasome, accelerating periodontal degradation [100]. Moreover, OS can impair antioxidant pathways by inhibiting the Nrf2 transcription factor, which regulates the expression of antioxidant genes [71]. Excessive ROS overload leads to decreased production of antioxidant enzymes, compromising defense mechanisms and favoring periodontitis progression.

### 4.5. Interaction Between Free Radicals and the Oral Microbiota

Recent advances in metagenomic analyses have reinforced the notion that common oral diseases, including dental caries and periodontitis, are polymicrobial in nature rather than resulting from a single pathogenic species [101]. Furthermore, comparative studies investigating the associations between oral microbiota composition and salivary OS markers have highlighted marked inter-individual variability [102]. These findings suggest that no specific microorganism can be consistently linked to ROS generation in the oral cavity. Such discrepancies may stem from variations in microbial community structure, site-specific colonization, and host immune responses, offering a plausible explanation for the heterogeneous data regarding the relationship between OS and oral inflammation [103].

The interaction between free radicals and the oral microbiota plays an essential role in maintaining the balance between oral health and pathology. Normally, ROS participate in immune responses and contribute to microbiome homeostasis. However, an excess of ROS can promote dysbiosis, amplifying inflammatory processes and favoring the progression of both oral and systemic diseases [104].

The oral microbiome is a complex ecosystem where microorganisms coexist in synergistic and antagonistic relationships. These interactions are regulated by competition for nutrients, the production of antimicrobial substances (e.g., nisin), nitrate metabolism, and pH control [105]. Excessive ROS production destabilizes this balance, promoting the development of caries and periodontitis.

*S. mutans*, a bacterium implicated in caries development, can produce reactive oxygen species (ROS), contributing to the progression of carious lesions. This oxidative activity is modulated by interactions with other microbial species, which may either enhance or suppress ROS generation. Moreover, *Fusobacterium nucleatum*, commonly found in both the oral cavity and the gastrointestinal tract, has been associated with systemic inflammatory conditions and colorectal cancer through mechanisms involving immune modulation, epithelial barrier disruption, and oxidative stress. These findings suggest a more specific role for oral bacteria in the pathogenesis of systemic diseases via oxidative mechanisms [106].

A clear example of the influence of OS on the oral microbiota is periodontitis. Chronic inflammation induced by ROS leads to the degradation of the periodontal ligament and alveolar bone. *P. gingivalis*, a bacterium strongly associated with periodontitis, thrives in a pro-inflammatory environment and accelerates tissue destruction [107]. The impact of ROS is not limited to the oral cavity, as periodontitis-associated inflammation and OS have been correlated with systemic conditions such as atherosclerosis, Alzheimer’s disease, and diabetes [108].

Moreover, free radicals affect the structure of the oral biofilm, promoting the growth of opportunistic pathogens and destabilizing the microbial ecosystem. These effects are mediated through the oxidation of biofilm components, modification of cellular receptor expression, and alteration of bacterial adhesion mechanisms. Additionally, the disruption of microbial coagulation processes compromises the integrity of the epithelial barrier, facilitating pathogen infiltration and contributing to the spread of inflammation. Furthermore, oral dysbiosis associated with increased ROS levels may influence the gut microbiota, with implications for systemic health [106].

## 5. Impact of OS on Oral Health

It has been demonstrated that OS can cause structural changes in the salivary glands, leading to decreased saliva secretion and alterations in the biochemical composition of saliva [66,109].

The World Health Organization has estimated that nearly 3.5 billion people worldwide are affected by oral diseases. Among the most common and severe conditions are dental caries, periodontal disease, and tooth loss, as well as malignant diseases of the lips and oral cavity Although not a panacea, phytotherapy is increasingly valued as a treatment method, offering a wide range of products for various diseases of the oral cavity.

### 5.1. Oral Aphthae

Oral aphthae are painful oral lesions characterized by small, round ulcers with a grayish color and an inflammatory halo. They can have viral, allergic, or traumatic causes and are frequently located on the tongue, inner cheeks, or lips [110].

OS plays a crucial role in the onset and progression of oral aphthae, promoting inflammation and delaying the healing process. ROS contribute to damage to cell membranes, proteins, and DNA, compromising the oral epithelial barrier and making tissues more vulnerable to infections and lesions. Additionally, OS can amplify the inflammatory response by activating pro-inflammatory cytokines (IL-1β, TNF-α) and reducing levels of endogenous antioxidants, such as GSH and SOD [111].

Individuals exposed to OS-inducing factors, such as smoking, alcohol consumption, unbalanced diets, or deficiencies in essential vitamins and minerals (especially vitamin C, E, and zinc), have a higher risk of developing oral aphthae. Furthermore, physical and emotional stress can disrupt redox balance and weaken the natural defense mechanisms of the oral mucosa, increasing the recurrence of aphthous ulcers [112].

### 5.2. Dental Abscesses

A dental abscess is a localized infection at the base of the tooth, characterized by swelling and deformation of the affected area. It presents with gingival inflammation, redness, congestion, and intense pain, especially upon pressure. In addition to bacterial factors, OS plays a significant role in the pathogenesis of dental abscesses, contributing to tissue damage and worsening inflammation [113].

During an infectious process, immune system cells release ROS as a defense mechanism against pathogenic bacteria. However, excessive ROS accumulation leads to oxidative damage in adjacent tissues. Furthermore, ROS can activate inflammatory pathways such as NF-κB, amplifying the secretion of pro-inflammatory cytokines (IL-1β, IL-6, TNF-α), which exacerbate swelling and local pain [114]. Oxidative damage to collagen and other extracellular matrix components impairs tissue regeneration, favoring infection spread and delayed healing. Additionally, OS increases vascular permeability and leukocyte migration, leading to excessive pus accumulation in the affected area [115].

### 5.3. Periodontitis and Gingivitis

ROS significantly contribute to collagen degradation and chronic gingival inflammation, accelerating the loss of tooth-supporting tissues. Periodontal diseases, such as gingivitis and periodontitis, are inflammatory conditions caused by subgingival bacterial biofilms, with OS being a key factor in their progression. During the immune response triggered by pathogens, polymorphonuclear cells release ROS, leading to the degradation of the extracellular matrix, periodontal ligaments, gingiva, and alveolar bone [116,117].

Excess ROS initiate lipid peroxidation and fatty acid oxidation, stimulating adipogenesis, inhibiting osteoblast formation, and activating osteoclasts, thereby promoting bone resorption. On a molecular level, this process is reflected by increased RANKL expression, which is essential for osteoclast activation, and enhanced TNF-α-mediated inflammation [118].

Patients with periodontitis show elevated levels of OS biomarkers, such as MDA and H_2_O_2_, which are correlated with reduced activity of antioxidant enzymes like SOD and CAT [100]. This imbalance creates a vicious cycle where ROS stimulate and osteoclast activation, worsening alveolar bone resorption and disease progression.

Lipopolysaccharides released by Gram-negative bacteria in the subgingival biofilm trigger a strong inflammatory response, promoting the secretion of pro-inflammatory cytokines, amplifying OS, and destroying periodontal tissue. ROS also act as intracellular signaling molecules, activating the NLRP3 inflammasome and promoting osteoclast differentiation, further contributing to bone resorption and chronic inflammation [119,120].

The oxidative imbalance in periodontal diseases not only worsens local tissue destruction but is also linked to systemic effects, including reduced insulin sensitivity and damage to other organs, playing a significant role in the pathogenesis of metabolic, cardiovascular, and neurological diseases.

### 5.4. Dental Caries

The development of dental caries is influenced by both OS and bacterial infections, which contribute to enamel demineralization by depleting essential ions such as calcium (Ca^2+^), magnesium (Mg^2+^), and phosphate (PO_4_^3−^). Cariogenic bacteria, particularly *Streptococcus* and *Lactobacillus*, produce acids that lower the pH below the critical threshold of 5.2–5.5, accelerating enamel degradation. At the same time, OS disrupts the antioxidant balance of the saliva, reducing TAC and promoting mineral loss, which is essential for maintaining enamel integrity [121].

*S. mutans*, one of the primary bacteria involved in caries, utilizes Spx regulators that confer resistance to OS, facilitating its survival and colonization in the oral cavity. Additionally, OS affects metal ion metabolism and the solubility of HA, the primary component of dental enamel. Under acidic conditions, HA’s solubility increases exponentially, accelerating mineral loss from the tooth structure [122,123,124].

Studies show that caries severity is correlated with an imbalance between pro-oxidant and antioxidant systems. Children with severe caries exhibit increased levels of TAC, SOD, UA, MDA, and GSH, alongside reduced NO levels, indicating a direct link between dental damage and antioxidant activity. Additionally, age influences this relationship: children have higher TAC levels than adolescents, and *S. mutans*, with high acidogenic activity, is more prevalent in the early stages of caries [125].

### 5.5. Precancerous Oral Lesions and Oral Cancer

Oral leukoplakia is the most common potentially malignant disorder of the oral mucosa, classified into homogeneous and non-homogeneous lesions, being more prevalent among the elderly and in certain regions, such as India. Early identification is essential, as many cases of oral cancer are preceded by such lesions [126,127].

OSCC accounts for over 90% of oral cancer cases, with approximately 300,000 cases globally per year. Often diagnosed late, it has a poor prognosis. Risk factors include smoking, alcohol consumption, HPV infection, and exposure to radiation and chemicals, as well as genetic predisposition. Clinically, OSCC presents as a persistent ulcerative lesion on the tongue, the floor of the mouth, or the lower lip.

Recent studies highlight the role of OS in OSCC development, showing an increase in free radicals and lipid peroxidation, along with a decrease in antioxidant levels, as factors that promote disease progression [128].

### 5.6. Xerostomia (Dry Mouth)

Factors such as oral microorganisms, alcohol consumption, tobacco use, medications, and fluoride exposure trigger pro-inflammatory cytokines, affecting salivary flow and composition. This leads to increased ROS production and oxidative damage in the oral cavity [129,130].

Certain medications, such as chemotherapeutic agents (cyclophosphamide, 5-fluorouracil), antibiotics (metronidazole), and analgesics (tramadol), induce OS in the salivary glands. These medications cause damage that leads to glandular atrophy and reduced saliva production, contributing to the development of xerostomia [131].

Chemicals like alcohol, heavy metals, and fluoride also induce OS in the salivary glands. These exposures can lead to histological changes, including parenchymal atrophy and increased fibrosis, contributing to reduced saliva production and, consequently, xerostomia [132].

## 6. Components of the Salivary Antioxidant System (Enzymatic and Non-Enzymatic)

In healthy individuals, small amounts of ROS, such as •OH, O_2_^−^, H_2_O_2_, and ONOO^−^, are efficiently neutralized by endogenous antioxidant systems. However, under pathological conditions, excessive ROS production surpasses the antioxidant neutralization capacity, leading to OS and disrupting redox balance [133].

To counteract the harmful effects of ROS, the body employs a complex antioxidant system, composed of two main categories: enzymatic and non-enzymatic. Enzymatic antioxidants include GSH peroxidase, myeloperoxidase, SOD, and CAT, which play a crucial role in neutralizing ROS. On the other hand, non-enzymatic antioxidants, such as minerals, vitamins, polyphenols, and thiols, contribute to maintaining redox balance and protecting cellular structures from OS [134].

In the oral cavity, the salivary antioxidant system is an essential component of the defense mechanism against OS. It includes a combination of enzymatic antioxidants and non-enzymatic antioxidant molecules, each playing a specific role in protecting oral tissues from ROS-induced damage.

### 6.1. Enzymatic Antioxidants in the Oral Cavity

Enzymatic antioxidants in the oral cavity neutralize free radicals before they damage cellular components. They reduce the energy of radicals or donate electrons to stabilize them. Additionally, they interrupt oxidative chain reactions, thereby limiting damage [9]. This category includes SOD, CAT, and GPx.

SOD is an essential antioxidant enzyme that protects cells against OS and prevents lipid peroxidation. In mammals, SOD has three main isoforms: SOD1 (Cu/Zn-SOD), located in the cytoplasm and dependent on copper and zinc; SOD2 (Mn-SOD), situated in the mitochondria and utilizing manganese as a cofactor; and SOD3 (EC-SOD), an extracellular enzyme present in biological fluids, including saliva [135].

In the oral cavity, SOD3 protects tissues by converting superoxide radicals into H_2_O_2_ and molecular oxygen. The resulting H_2_O_2_ is then detoxified by CAT and GPx, completing the antioxidant defense system [136]. The high specificity of SOD for O_2_^−^ prevents unwanted interactions with other molecules, such as NO, which is involved in cellular regulation [137].

SOD2, localized exclusively in the mitochondria, is the first line of defense against mitochondrial OS. Beyond ROS protection, SOD2 regulates mitochondrial autophagy and the JAK/STAT and PI3K/Akt signaling pathways [77]. Its expression significantly increases in periodontitis, correlating with disease severity, especially in diabetic patients. Infection with *P. gingivalis* and *Fusobacterium nucleatum* activates the inflammatory response in oral epithelial cells, increasing OS and stimulating the NF-κB pathway, which regulates SOD2 expression [138,139]. With aging, SOD2 expression decreases, while activation of the mTOR/AKT pathway promotes mitochondrial dysfunction and chronic inflammation [139].

SOD2 regulates apoptosis in a manner depending on the cell type. In immune cells, it promotes apoptosis, contributing to the inflammatory response. In periodontal cells, it has a protective effect, maintaining tissue integrity. SOD2 stimulates the osteogenic differentiation of periodontal ligament stem cells (hPDLSCs), facilitating alveolar bone regeneration [140,141]. Thus, SOD2 plays a dual role in periodontitis—protecting fibroblasts and epithelial cells but inducing apoptosis in immune cells, influencing disease progression.

In oral cancer, SOD2 acts both as a tumor suppressor and a tumor promoter, depending on the disease stage. In the early stages, SOD2 inhibition leads to excessive ROS accumulation, favoring DNA mutations and cellular transformation. In the advanced stages, SOD2 overexpression leads to H_2_O_2_ accumulation, activating signaling pathways (ERK1/2-Slug) that promote epithelial–mesenchymal transition, migration, and metastasis [142,143,144].

Moreover, SOD2 levels are significantly increased in patients with dental caries and irreversible pulpitis, correlating positively with disease severity [145].

Monitoring SOD levels has diagnostic and prognostic potential in oral conditions. In periodontitis, SOD regulates inflammation and redox balance; in oral cancer, it plays a dual role in carcinogenesis; in dental caries and pulpitis, it indicates the activation of the antioxidant response; and in oral submucous fibrosis, it can be a biomarker of OS induced by betel quid and tobacco.

SOD levels are significantly reduced in patients with OSCC, attributed to free radical accumulation and increased SOD consumption [146]. Some studies report increased SOD activity in lymphocytes and saliva, suggesting a compensatory response to OS [147,148].

CAT is a key enzyme in cellular defense, catalyzing the breakdown of H_2_O_2_ into water and molecular oxygen. This reaction prevents toxic H_2_O_2_ accumulation and complements SOD detoxification. Due to its exceptional catalytic efficiency, CAT can degrade millions of H_2_O_2_ molecules per second [149].

The tetrameric structure of CAT contains iron-rich heme groups, and its predominant localization in peroxisomes allows for the neutralization of H_2_O_2_ generated via metabolic processes such as fatty acid β-oxidation. In mammalian mitochondria (except for rat heart mitochondria), H_2_O_2_ detoxification is performed by GPx, indicating a complementary distribution of antioxidant systems [150].

The catalytic mechanism of CAT occurs in two stages: in the first stage, H_2_O_2_ oxidizes the heme group, forming a radical oxoferryl porphyrinic intermediate (Compound I); in the second stage, another H_2_O_2_ molecule reduces the intermediate, regenerating the enzyme and releasing water and oxygen [151]. Beyond its primary function, CAT can also decompose ONOO^−^ and NO, playing a broader role in cellular redox balance [152].

CAT is involved in immune response activation, cell proliferation, and apoptosis. It also exhibits peroxidase activity, oxidizing compounds such as ethanol and phenols, giving it metabolic versatility [149]. CAT deficiency can affect the viability of mesenchymal stem cells [153].

There are three main classes of CAT: typical heme-containing CAT, predominant in mammals; catalase-peroxidases; and manganese-containing CATs, found in bacteria and fungi. Typical CAT exhibits the highest enzymatic activity, while the other types have lower catalytic efficiencies [154].

GPx is a family of selenium-containing antioxidant enzymes that neutralize lipid hydroperoxides and H_2_O_2_. The selenium atom in its structure makes GPx essential for cellular defense against OS [155].

Eight GPx isoforms (GPx1–GPx8) have been identified, each with specific functions in cellular protection. GPx-1, the most abundant isoenzyme, protects mitochondria, regulates cell signaling, and prevents DNA mutations, potentially exhibiting anticancer effects. By eliminating hydroperoxides, GPx contributes to redox balance maintenance and the prevention of oxidative damage. The GPx enzymatic system also includes other homologs, such as iodide peroxidase, myeloperoxidase, and hydrogen peroxidase, which act synergistically to maintain cellular homeostasis [155].

In the oral cavity, salivary GPx plays a crucial role in tissue protection by regulating the levels of H_2_O_2_ released by salivary glands, bacteria, and leukocytes. It catalyzes the oxidation of bromide, thiocyanate, and iodide ions, reducing H_2_O_2_ to water while generating antibacterial compounds. Additionally, GPx contributes to the formation of hypochlorous acid and chloramines, which protect the oral cavity against pathogens [156].

Lactoperoxidase (LPO) is one of the most important enzymatic antioxidants in human saliva, playing a crucial role in maintaining microbial balance and protecting against pathogens. Together with myeloperoxidase (MPO), which is secreted by neutrophils in the gingival space, it forms the oral peroxidase system (OP), which contributes to the neutralization of oral bacteria and the reduction in H_2_O_2_ levels produced by the oral microbiota [157].

The mechanism of action of LPO and MPO involves the oxidation of thiocyanate ions (SCN^−^), derived from the diet, in the presence of H_2_O_2_, generating hypothiocyanite ions (OSCN^−^)—compounds with strong antimicrobial effects. These ions inhibit essential bacterial enzymes and disrupt microbial membrane integrity, thereby reducing the proliferation of pathogenic microorganisms in the oral cavity [158].

MPO, in addition to its antimicrobial role, is released into saliva through the lysis of neutrophils in the gingival sulcus, along with other antimicrobial proteins such as lysozyme and lactoferrin. This enzyme catalyzes the conversion of H_2_O_2_ into reactive species that are capable of destroying oral bacteria. Additionally, salivary peroxidases help to neutralize H_2_O_2_ produced by bacteria and epithelial cells, potentially playing a role in the inactivation of toxic substances with carcinogenic, mutagenic, and genotoxic potential. Through these mechanisms, the oral peroxidase system plays an essential role in maintaining oral health, protecting tissues from OS, reducing inflammation caused by microbial activity, and preventing the harmful effects of oxidative substances [159].

Lactoferrin is a cationic glycoprotein that is primarily secreted by glandular epithelia and neutrophils, possessing multiple essential physiological functions due to its anti-infective, immunomodulatory, and antioxidant properties. Initially, its role was believed to be solely related to iron-binding capacity; however, later research has demonstrated a pleiotropic action, involving multiple defense mechanisms of the body [160].

In the oral cavity, lactoferrin plays a central role in maintaining microbial balance and protecting against infections. Its levels are influenced by inflammatory and microbiological changes associated with periodontitis, making it a potential diagnostic biomarker for periodontal disease. Beyond its diagnostic value, lactoferrin has adjunctive therapeutic benefits in periodontal treatment, through its interactions with local microorganisms, anti-inflammatory effects, and antioxidant activity [161].

### 6.2. Non-Enzymatic Antioxidants

These are compounds with a direct role in neutralizing free radicals and include uric acid (UA), GSH, vitamin C (ascorbic acid), vitamin E (α-tocopherol), albumin, polyphenols, selenium, and carotenoids.

UA, the final product of purine metabolism, is one of the most abundant antioxidants in the human body, protecting against carcinogenesis by neutralizing free radicals. It is the primary antioxidant present in saliva, accounting for over 70% of its total antioxidant capacity in both stimulated and unstimulated saliva [162]. UA plays a crucial role in defending the body against OS and free radicals, contributing to redox balance and oral tissue protection. Recent studies indicate that the TAC of saliva increases with the severity of dental caries, suggesting that antioxidant mechanisms are activated in response to inflammatory processes and oral tissue damage [163]. Clinical studies have demonstrated that decreased salivary UA levels are associated with various oral pathologies characterized by elevated OS, such as periodontitis, oral lichen planus, and OSSC. For instance, patients with OSSC exhibited significantly lower salivary UA concentrations compared to healthy individuals, indicating a compromised antioxidant defense in the oral environment [164]. Furthermore, research indicates that salivary UA levels can reflect systemic OS conditions. In cases of metabolic syndrome, elevated salivary UA concentrations have been observed, correlating with increased OS markers [162]. Additionally, in cases of severe dental caries in children, salivary TAC was higher but negatively correlated with salivary pH, suggesting that OS influences the chemical balance of saliva and contributes to the progression of oral lesions [165].

GSH is an essential cellular antioxidant that is involved in maintaining redox balance and protecting against OS. It is found in high concentrations in the cytosol, mitochondria, and nucleus, where it neutralizes ROS such as •OH, H_2_O_2_, and singlet ^1^O_2_ [166].

Beyond its antioxidant function, GSH contributes to the regeneration of vitamins C and E, thus protecting biomolecules from oxidative damage. It also supports DNA stability and repair by maintaining the sulfhydryl (-SH) groups of cellular proteins. Without this mechanism, proteins may undergo irreversible modifications, affecting cellular functionality [167]. Additionally, sulfur-containing compounds in GSH contribute to free radical neutralization and protect biomolecules from OS

The GSH/GSSG ratio is a fundamental indicator of cellular antioxidant capacity, since GSH’s oxidation to glutathione disulfide (GSSG) under the influence of free radicals can impact cellular homeostasis [168]. GSH regeneration is supported by GPx, while glutathione reductase (GR), encoded by the *GSR* gene, ensures the conversion of GSSG to GSH, maintaining an optimal ratio necessary for protection against OS [169].

At the oral level, GSH plays a key role in protecting tissues from lipid peroxidation and detoxifying reactive oxygen species. Studies have shown that GSH levels are reduced in chronic oral infections, such as periodontitis, indicating the depletion of antioxidant mechanisms and increased susceptibility to inflammation and periodontal tissue destruction [170,171].

Regarding dental caries, research by [172] highlighted a negative correlation between salivary GSH concentration and caries severity, suggesting a potential compensatory mechanism in the early stages of the disease. However, in the advanced stages, increased antioxidant consumption may lead to depletion, promoting the progressive deterioration of dental tissues [173].

Therefore, GSH plays a crucial role in maintaining oral health and preventing inflammatory and degenerative diseases, acting as an essential factor in OS protection and cellular homeostasis maintenance.

Vitamin C (ascorbic acid) is a water-soluble antioxidant that is essential for neutralizing free radicals and regenerating other antioxidants, such as vitamin E. Under physiological conditions, it acts through the ascorbate anion (AscH^−^), which interacts with free radicals (R•), forming the ascorbyl radical (Asc•^−^), a compound with minimal oxidative effects. This property allows ascorbic acid to protect cell membranes by neutralizing peroxyl radicals before they oxidize lipids, providing an effective defense against OS [60].

Vitamin C contributes to redox balance maintenance and prevents oxidative damage to cells. It also plays a vital role in regenerating vitamin E from its radical form (tocopheryl radical), strengthening the body’s antioxidant mechanisms. In cases of nutritional deficiency or chronic inflammation, its concentration decreases, which may compromise the cellular defense against OS. Under certain conditions, vitamin C may exhibit pro-oxidant effects, particularly in the presence of free transition metals such as iron and copper, which generate ROS and promote OS. However, in the body, metals’ metabolism is tightly regulated, and their concentrations are maintained at very low levels, limiting the pro-oxidant effects of vitamin C under physiological conditions [174].

Vitamin E (α-tocopherol) is a liposoluble antioxidant that is essential for protecting biological membranes against lipid peroxidation caused by ROS. It anchors in cell membranes, where it interacts with free radicals, neutralizing them and preventing cellular damage. It acts as a peroxyl radical scavenger by donating a hydrogen atom, converting the peroxyl radical into a neutral species and stopping the propagation of OS [75]. The resulting tocopheroxyl radical is regenerated by other antioxidants, such as vitamin C, maintaining the active α-tocopherol form and ensuring a synergistic antioxidant protection mechanism [60].

Ferroptosis is a regulated form of cell death characterized by iron-dependent accumulation of lipid peroxides at lethal levels. Studies have demonstrated that vitamin E, including forms such as α-tocopherol and tocotrienols, acts as an effective lipophilic antioxidant, protecting cells against uncontrolled lipid peroxidation, and thereby preventing the induction of ferroptosis [175].

Albumin is a salivary protein with a crucial role in protection against OS, capable of binding metal ions such as iron and copper. Through this interaction, it prevents these metals from participating in the Fenton reaction, which generates highly reactive and harmful hydroxyl radicals [176]. In addition to its antioxidant function, albumin contributes to stabilizing salivary pH and protecting dental enamel, maintaining the integrity of the oral cavity.

Polyphenols are secondary metabolites of plants, with over 8000 representatives, produced in response to ultraviolet radiation and pathogen aggression [177]. In the oral cavity, they play a significant role in combating OS by neutralizing ROS, preventing lipid peroxidation, modulating antioxidant enzymes, protecting against inflammation and oral diseases, and inhibiting bacterial growth and biofilm formation.

Selenium is an essential element and a key component of selenoproteins, which play an antioxidant role and protect cells from ROS-induced damage. Through these proteins, selenium contributes to numerous essential biological processes, offering multiple health benefits. Studies have shown that adequate selenium intake can reduce the risk of cancer by up to 30%. Additionally, it supports cardiovascular health by protecting cell membranes and maintaining normal platelet function. However, selenium deficiency and excess have both been associated with an increased risk of cardiovascular diseases [178].

Carotenoids are natural pigments that are found in plants, bacteria, and algae, recognized for their antioxidant properties and health benefits, including the prevention of cancer, macular degeneration, and atherosclerosis [179]. Their antioxidant capacity derives from their chemical structure, rich in conjugated double bonds, which stabilize unpaired electrons and neutralize free radicals, protecting cell membranes from oxidative damage. These compounds can effectively scavenge ROO•, RO•, •OH, and O_2_^−^ through mechanisms such as hydrogen abstraction, electron transfer, and radical addition, thereby maintaining cellular redox balance [180].

## 7. Medicinal Plants with Antioxidant Potential from the Lamiaceae and Asteraceae Families

### 7.1. Bioactive Compounds with Antioxidant Roles

Antioxidants from medicinal plants play a crucial role in the food and pharmaceutical industries due to their ability to prevent and treat various conditions. Medicinal and aromatic species, particularly those traditionally used in ethnopharmacology, are recognized for their high contents of bioactive compounds with antioxidant properties [181]. These compounds can be regarded as abundant sources of allelochemicals. These results are most likely due to a synergistic interaction between the primary bioactive constituents (phenols) and other chemical components present in the extracts. Over time, medicinal plants have been appreciated as safe and effective sources of natural antioxidants, capable of neutralizing free radicals and protecting cells against OS [182]. Polyphenols are bioactive compounds that are present in plants, contributing to their color, taste, and aroma [183]. They are classified into flavonoids, phenolic acids, lignans, tannins, and stilbenes, with beneficial effects on health and the ability to reduce OS [184]. Interest in polyphenols is due to their use in food preservation and medical therapies [185], as well as their antioxidant, antibacterial, and anti-inflammatory properties [186]. Thus, they can contribute to the prevention of chronic diseases, including oral conditions.

Polyphenols protect cellular membrane lipids from degradation caused by free radicals, inhibiting lipid peroxidation and preventing the formation of toxic compounds such as MDA and 4-hydroxy-2-nonenal (4-HNE) [187,188].

In the oral cavity, polyphenols interact with salivary glycoproteins, causing an astringent and bitter taste in fruits and juices due to the presence of procyanidins [189,190]. Being soluble in saliva, they act on the oral mucosa and salivary proteins [191]. Polyphenols inhibit bacterial biofilm formation, reduce dental plaque, and contribute to oral hygiene [192]. In the case of halitosis, they reduce volatile sulfur compounds (VSCs), the primary cause of bad breath.

The antioxidant mechanisms of polyphenols include free radical scavenging through hydrogen atom or electron transfer, as well as chelation of transition metals. In oral cancers, polyphenols inhibit tumor cell proliferation and reduce migration and invasion [193].

#### 7.1.1. Phenolic Acids

Phenolic acids are natural compounds with antioxidant properties found in plants and used to prevent oxidative damage due to their solubility in both lipids and water. Their antioxidant activity is determined by their chemical structure, with the main mechanism being the donation of hydrogen atoms to neutralize free radicals [194]. Gallic acid (GA), a powerful antioxidant, is widely distributed in plants and used in the food and pharmaceutical industries [195]. Rosmarinic acid, a natural polyphenol present in plants such as rosemary (*Rosmarinus officinalis*), sage (*Salvia officinalis*), and thyme (*Thymus vulgaris*), has antioxidant, anti-inflammatory, and antimicrobial properties. Incorporating gallic acid, rosmarinic acid, or plant extracts containing them into oral hygiene products could provide additional benefits for oral health. In a study on species from the Lamiaceae family using Fourier-transform infrared spectroscopy (DRIFTS), the concentration of rosmarinic acid ranged from 81 ± 4 mg/g in lemon balm to 12 ± 3 mg/g in oregano from dry plant material [196].

#### 7.1.2. Flavonoids

Flavonoids are a class of phenolic compounds with low molecular weight, with variable structures. They are divided into six main categories: flavones (e.g., apigenin, luteolin), flavonols (e.g., quercetin, myricetin), flavanones (e.g., naringenin, hesperidin), catechins or flavanols (e.g., epicatechin, gallocatechin), anthocyanidins (e.g., cyanidin), and isoflavones (e.g., genistein, daidzein). Widely distributed in the plant kingdom, flavonoids are found in fruits, roots, stems, flowers, and bark, as well as in plant-derived foods and beverages such as tea, cocoa, wine, and cereals, where they are called dietary flavonoids [197]. They are associated with multiple health benefits and have applications in the pharmaceutical, medicinal, cosmetic, and nutraceutical fields due to their antioxidant properties [198]. Studies have shown that flavones are the most effective in protecting the body against ROS [199]. Furthermore, the antioxidant capacity of flavonoids varies depending on their chemical structure, with each compound having a specific mechanism of action while offering effective protection against OS [200].

#### 7.1.3. Tannins

Tannins are an important subgroup of polyphenolic compounds, divided into hydrolyzable tannins and condensed tannins. They are widespread in plants and are widely used in the food and medical fields due to their antioxidant, antibacterial, and anti-inflammatory properties [201]. Recent studies have highlighted their role in preventing cardiovascular diseases, cancer, and osteoporosis, which has led to increased interest in biomedical research.

Tannins are known for their astringent effect, which influences various biological activities similar to those of flavonoids. The antibacterial activity of tannins is attributed to their interaction with bacterial proteins, destabilizing cell membranes and inhibiting the growth of pathogenic microorganisms. In the oral cavity, tannins interact with salivary proteins, especially with histidine residues, leading to protein precipitation and reduced natural lubrication. This reaction is responsible for the sensation of dryness and contraction of the oral mucosa, characteristic of the astringent taste experienced when consuming tannin-rich foods [202].

#### 7.1.4. Essential Oils

Essential oils are volatile compounds with biological activity, playing an important role in combating OS. They primarily contain mono-, sesqui-, and diterpenoids, phenols, and other active compounds, and they are obtained through steam distillation or pressing [203]. Recently, the antioxidant potential of essential oils has been highlighted, with their ability to prevent oxidative damage caused by free radicals. Their mechanism of action involves donating hydrogen atoms from phenolic hydroxyl groups to neutralize peroxyl radicals, thus slowing down the peroxidation of unsaturated lipids [204]. Eugenol and thymol, major components of essential oils, inhibit the propagation of oxidative reactions through this mechanism, while linalool and menthol contribute to the self-termination and cross-linking of oxidative chains [205,206,207]. Due to these properties, essential oils extracted from *Syzygium aromaticum*, *Salvia officinalis*, *Matricaria chamomilla*, *Mentha piperita*, *Thymus serpyllum*, *Eucalyptus*, *Melissa officinalis*, *Foeniculum vulgare*, and *Cinnamomum cassia* are used in dentistry for their antioxidant and anti-inflammatory effects [208]. They help protect oral structures from OS, playing a significant role in preventing cellular damage caused by reactive oxygen species.

#### 7.1.5. Anthocyanins

Anthocyanins are natural pigments that are responsible for the red, purple, and blue colors of flowers, fruits, leaves, and roots, with the ability to change their hue depending on cellular pH. About one-third of flavonoids are anthocyanins, recognized for their antioxidant properties and ability to neutralize free radicals. They contribute to protection against OS by reducing permeability and increasing capillary resistance. Additionally, they inhibit platelet aggregation, promote clot retraction, and exert anti-inflammatory effects. Through their diuretic activity, they support the elimination of uric acid, playing a beneficial role in maintaining redox balance and preventing oxidative damage to oral tissues [209].

#### 7.1.6. Terpenes

Terpenes are an extensive class of natural compounds with antioxidant properties that are essential for protection against OS. They neutralize free radicals, preventing cellular damage and protecting essential biomolecules such as lipids, proteins, and DNA from peroxidation and oxidative degradation. Their antioxidant mechanism relies on three main processes: free radical scavenging through electron donation, stimulation of endogenous antioxidant enzymes such as SOD and CAT, and maintenance of the integrity of essential biomolecules. Due to these effects, terpenes play an important role in reducing inflammation, modulating immune responses, and protecting against diseases associated with OS, including cardiovascular and neurodegenerative diseases, cancer, and diabetes [210].

#### 7.1.7. Alkaloids

Alkaloids are natural compounds with antioxidant, antibacterial, and anti-inflammatory properties, playing an important role in protection against OS. They inhibit collagen degradation and reduce the effects of free radicals, thus contributing to the maintenance of oral health. A relevant example is berberine, which exhibits antibacterial activity against *Aggregatibacter actinomycetemcomitans* and *Porphyromonas gingivalis*, species involved in periodontal disease. Its mechanism of action consists of inhibiting collagenase enzymes, protecting the structure of connective tissues [211]. Due to their chemical structure containing a nitrogen atom, alkaloids act as redox agents, preventing the oxidation of proteins and lipids. Additionally, they influence signaling pathways involved in inflammation, showing significant therapeutic potential. However, their use requires caution, as some may have toxic effects at high doses [212].

### 7.2. Sources of Natural Antioxidants from Medicinal Species of the Asteraceae and Lamiaceae Families

#### 7.2.1. Plants from the Lamiaceae Family

The Lamiaceae family includes numerous plants with antioxidant potential and medical uses due to their rich contents of flavonoids, polyphenols, essential oils, and phenolic acids—compounds with antioxidant and anti-inflammatory activities [213,214]. The therapeutic effectiveness of these plants depends on factors such as climate, altitude, sun exposure, conservation method, and preparation process. Active compounds contribute to cellular protection against OS and support natural healing processes, being used in dietary supplements, cosmetics, and medicines. Today, many widely used drugs are synthetic analogs of secondary plant metabolites, demonstrating the importance of these compounds in the development of modern therapies [215,216,217,218]. The relevant species from the Asteraceae family and their beneficial effects are presented in Table 1.

#### 7.2.2. Plant Species of the Asteraceae Family with Antioxidant Constituents

The Asteraceae family is one of the largest and most diverse plant families, including numerous species with medicinal potential. These plants are valued for their high contents of antioxidant compounds, which help combat OS, including in the oral cavity. Due to their antioxidant, anti-inflammatory, and antimicrobial properties, plants from this family can support both oral and general health [283].

The relevant species from the Asteraceae family and their beneficial effects are presented in Table 2.

## 8. Medicinal Plants from the Lamiaceae and Asteraceae Families in Modulating Salivary Antioxidant Defense

Medicinal plants from the Lamiaceae and Asteraceae families are recognized for their rich contents of bioactive antioxidant compounds, which play an important role in modulating salivary antioxidant defense. These compounds help protect the oral cavity from OS, inflammation, and infections.

### 8.1. Lamiaceae Family

Species from the Lamiaceae family are essential for oral health maintenance due to their antioxidant, anti-inflammatory, and antimicrobial effects. The bioactive compounds of these plants (flavonoids, polyphenols, and essential oils) help protect the oral cavity from OS, inflammation, and infections. They are used in mouthwashes, toothpastes, tinctures, and infusions for the prevention of dental caries, gingivitis, halitosis, and periodontitis. These medicinal plants can be included in oral hygiene routines as natural mouth rinses, teas, essential oils, and supplements for comprehensive oral cavity protection. The Lamiaceae family is one of the most important sources of medicinal plants with applications in dentistry [328].

#### 8.1.1. *Lavandula angustifolia* L.

*Lavandula angustifolia* is a valuable natural remedy in dentistry, used for both preventing dental conditions and reducing pain and anxiety during dental treatments [329].

Lavender contains flavonoids, coumarins, and polyphenols with strong antioxidant effects, neutralizing free radicals and preventing OS, which can affect gum and oral mucosal health. It also protects oral epithelial cells from premature aging and damage caused by inflammation or infections [330].

Lavender mouthwash helps maintain oral hygiene, reduce gingival inflammation, and prevent infections. Lavender essential oil has potent antiseptic properties against *Staphylococcus aureus* and *Enterococcus coli* [331]. When applied topically, it relieves dental pain and promotes the healing of aphthous ulcers.

A randomized, double-blind, placebo-controlled study demonstrated the efficacy of lavender oil in treating recurrent aphthous ulceration. The results showed a significant reduction in ulcer size, accelerated mucosal healing within 2–4 days, and pain relief from the first application, with no reported side effects [332].

#### 8.1.2. *Mentha* × *piperita* L.

Peppermint (*Mentha × piperita* L.) is a valuable natural remedy in dentistry, used for maintaining oral health and protecting against OS. It is a natural hybrid between *Mentha viridis* and *Mentha aquatica*, originating from the Mediterranean region.

Peppermint contains bioactive compounds with strong antioxidant effects, such as flavonoids, polyphenols, and rosmarinic acid, which neutralize free radicals and protect epithelial cells in the oral cavity against OS, premature aging, and damage caused by inflammation or infections [333,334]. Menthol, the main active component of peppermint, has a cooling and mild anesthetic effect, which might make it effective in relieving dental and gingival pain [335,336].

*Mentha × piperita* is frequently used in mouthwashes and oral gels to help reduce inflammation and pain in gingivitis and periodontitis. It also accelerates the healing of oral lesions such as aphthous ulcers, stomatitis, and oral mucositis. Due to its antibacterial properties and fresh aroma, peppermint is effective in combating halitosis. Through its beneficial effects on oral health, *Mentha × piperita* is an essential ingredient in dental hygiene products, contributing to cavity prevention, reducing gingival inflammation, and maintaining fresh breath [337].

#### 8.1.3. *Ocimum basilicum* L.

Basil (*Ocimum basilicum*) is a plant rich in antioxidant compounds, widely used in the food, cosmetics, and pharmaceutical industries due to its health benefits [338]. The phenolic compounds in basil act as hydrogen and electron donors, neutralizing free radicals and protecting cells against OS, a major factor in inflammation and chronic diseases, including oral conditions [339,340].

Its antioxidant properties are attributed to its high contents of flavonoids, polyphenols, and rosmarinic acid, which show promising results in reducing OS and maintaining the health of the gums and oral mucosa [341,342]. Additionally, essential oils from *O. basilicum* have antioxidant activity due to phenolic compounds, contributing to tissue protection against oxidative damage.

Due to its antimicrobial, antioxidant, and anti-inflammatory properties, basil presents potential as a remedy in dentistry, used in oral hygiene for the prevention and treatment of oral cavity disorders. Its applications include natural basil mouthwash, suggesting beneficial effects in reducing gingival inflammation and combating halitosis, oral gels for the treatment of aphthous ulcers and gingivitis, and basil essential oil, added to toothpaste for its antibacterial effects [243].

Thus, basil might represent a natural alternative to conventional oral hygiene products, contributing to the maintenance of oral cavity health and protection against OS.

#### 8.1.4. *Rosmarinus officinalis* L.

Rosemary (*Rosmarinus officinalis* L.) is a Mediterranean shrub that is used in dentistry for cavity prevention, reducing gingival inflammation, and protecting oral tissues against OS [231,343,344]. It contains flavonoids, polyphenols, and rosmarinic acid, bioactive compounds that neutralize free radicals and prevent premature aging of the oral mucosa [345].

Studies show that rosemary extracts indicate potential in preventing the harmful effects of OS on oral tissues and contribute to protection against chronic inflammation [344]. An in vitro study demonstrated the ability of *Rosmarinus officinalis* extract to eliminate initial microbial biofilms, supporting its use as an adjuvant in the treatment of periodontitis and cavities [345]. Rosemary essential oil contains cineole, camphor, carnosol, and rosmarinic acid, active compounds with antibacterial action against *S. mutans* and *P. gingivalis* [346]. Additionally, *Rosmarinus officinalis* reduces bacterial plaque and tartar formation and exhibits antifungal activity against *Candida albicans*, preventing oral infections [347]. A study conducted on Egyptian children showed that *R. officinalis* extract has antibacterial effects against *S. mutans*, proving to be safer than chlorhexidine due to its natural composition [348].

Due to its antiseptic effects and pleasant aroma, rosemary is used in mouthwashes and toothpastes to freshen breath. A randomized, double-blind study found that a toothpaste containing *Rosmarinus officinalis* extract significantly reduced gingival bleeding and plaque accumulation compared to conventional toothpastes [349].

A live animal study analyzed the effects of rosmarinic acid as a radioprotective agent for the parotid gland during radiotherapy. In an irradiated rat model, rosmarinic acid was shown to reduce OS, inflammation, and tissue fibrosis, protecting salivary secretion. Compared to amifostine, rosmarinic acid proved to be more effective in the long term and easier to administer, demonstrating potential as a radioprotective agent [350].

Rosemary is a valuable natural solution for maintaining oral health due to its antioxidant, antimicrobial, and anti-inflammatory properties. It is used in oral hygiene products to prevent cavities, periodontitis, and oral infections, while also offering protection against OS and mucosal damage.

#### 8.1.5. *Salvia officinalis* L.

Sage (*Salvia officinalis*) is a valuable plant in dentistry due to its antioxidant, antimicrobial, and anti-inflammatory properties. It contains polyphenols, flavonoids, and rosmarinic acid, compounds that neutralize free radicals and protect oral tissues from OS, preventing mucosal damage and slowing chronic inflammatory processes [337,351,352] Sage contains bioactive compounds such as rosmarinic acid, carnosol, and cineole, which act against pathogenic oral bacteria, including *S. mutans* and *P. gingivalis*. These compounds reduce bacterial plaque and tartar formation, protecting the gums and dental enamel from the harmful effects of bacterial toxins and OS [353]. Sage essential oil has a mild anesthetic effect, helping to relieve dental and gingival pain. It also has antifungal activity against *Candida albicans*, preventing oral infections and accelerating the healing of aphthous ulcers, lesions, and post-extraction wounds [354].

Used as infusions, tinctures, mouthwashes, or essential oil, sage significantly contributes to oral health. Studies so far have yielded encouraging findings in treating gingivitis, aphthous ulcers, and halitosis, as well as preventing cavities [353]. Additionally, due to its antibacterial and astringent effects, it helps eliminate the bacteria responsible for bad breath [355].

#### 8.1.6. *Satureja hortensis* L.

*Satureja hortensis* L., commonly known as summer savory, is an aromatic plant that is valued for its antioxidant and anti-inflammatory properties, with potential applications in dentistry for preventing cavities and reducing gingival inflammation [260]. It contains polyphenols, flavonoids, and rosmarinic acid, which neutralize free radicals and protect oral tissues from OS, preventing mucosal damage and slowing chronic inflammatory processes [356]. The essential oil of *Satureja hortensis* is rich in phenolic compounds, such as carvacrol and γ-terpinene, which help maintain the microbiological balance of the oral cavity [357]. By protecting the gums and tooth enamel from bacterial toxins and OS, summer savory supports oral health and tissue integrity [358].

Used in the form of infusions, tinctures, mouthwashes, or essential oil, *Satureja hortensis* is a natural remedy that is beneficial for oral care. Studies have demonstrated its efficacy in combating oral infections and treating stomatitis caused by *Candida albicans* [359,360].

#### 8.1.7. *Thymus serpyllum* L.

Wild thyme (*Thymus serpyllum* L.) is a medicinal plant with antimicrobial, anti-inflammatory, and antioxidant properties, used in dentistry for oral tissue protection and prevention of dental conditions. It contains flavonoids, phenolic acids, tannins, and terpenic compounds that neutralize free radicals and reduce OS in the oral cavity. The most important antioxidant compounds include thymol, carvacrol, luteolin, apigenin, and rosmarinic acid [361]. OS is a key factor in the development of gingivitis and periodontitis, and the antioxidants in wild thyme protect gingival collagen and reduce chronic inflammation. The use of wild thyme extracts or thyme mouthwash contributes to gingival health maintenance [362,363]. The essential oil of *Thymus serpyllum* is rich in thymol, a phenolic compound with antibacterial and antifungal activity. Studies have shown that *Thymus vulgaris* is effective against *Candida albicans*, a common cause of dental stomatitis and root canal infections [364].

Due to its antiseptic properties, wild thyme is beneficial in combating halitosis. One study demonstrated that using thyme mouthwash after periodontal treatment was effective in improving bad breath and reducing gingival inflammation [365]. Additionally, thymol is used as an active ingredient in temporary dental fillings [366].

Wild thyme is also a rich source of gallic acid (GA), a natural antioxidant with food and pharmaceutical applications. However, its bioavailability is influenced by factors such as the gut microbiota and metabolism [367,368]. Thus, *Thymus serpyllum* is a medicinal plant with multiple benefits for oral health, used in dental hygiene products and natural therapies against OS and oral infections.

#### 8.1.8. *Thymus vulgaris* L.

*Thymus vulgaris* L., commonly known as thyme, has been used since ancient times for its therapeutic benefits, attributed to its rich polyphenol contents, particularly thymol and carvacrol. These compounds provide antiseptic, anti-inflammatory, and tissue-protective properties [369,370,371,372]. In dentistry, *Thymus vulgaris* is used for preventing and treating oral infections, reducing gingival inflammation, and accelerating healing. The volatile oil of thyme is effective against *S. mutans* and *Candida albicans* [364,373]. Additionally, thyme extracts are used for the treatment of oral herpes and protection of the oral mucosa from OS [374].

### 8.2. Asteraceae Family

The Asteraceae (Compositae) family is one of the largest medicinal plant families, known for its antioxidant, anti-inflammatory, and antimicrobial properties. Species from this family are used in dentistry for gum protection, OS reduction, and prevention of oral conditions such as gingivitis, periodontitis, and stomatitis. These plants contribute to oral mucosal health and can be used in infusions, tinctures, mouth rinses, or extracts, providing a natural alternative for oral care.

#### 8.2.1. *Matricaria chamomilla* L.

Chamomile (*Matricaria chamomilla* L.) is a medicinal plant that is frequently used in dentistry due to its antioxidant, anti-inflammatory, and antimicrobial properties [199]. It contains flavonoids and polyphenols, which have the capacity to neutralize free radicals, protect oral tissues from OS, and help maintain the oral mucosa’s health [375]. Thanks to its active compounds, such as α-bisabolol and chamazulene, chamomile has strong anti-inflammatory effects, making it effective in treating gingivitis, stomatitis, and aphthous ulcers [376]. Studies have shown that *Matricaria chamomilla* extracts inhibit the growth of oral bacteria, including *S. mutans*, *P. gingivalis*, and *Candida albicans* [377,378]. Additionally, it accelerates the healing of oral lesions and burns caused by dental prosthetics or surgical interventions [379]. *Matricaria chamomilla* can be used in various forms to maintain oral health, such as mouthwash to reduce gingival inflammation, infusions for rinses or compresses in the treatment of aphthous ulcers and stomatitis, essential oil applied locally for antimicrobial and soothing effects, or gels and toothpastes for gum protection [380]. Thus, chamomile represents an effective natural alternative for the prevention and treatment of oral conditions.

#### 8.2.2. *Cichorium intybus* L.

*Cichorium intybus* L., commonly known as chicory, is a valuable natural source for maintaining oral health due to its antioxidant, anti-inflammatory, and antimicrobial properties [381]. It contains bioactive compounds, such as polyphenols, flavonoids, and inulin, which has been shown to reduce free radicals and protect the oral mucosa and gingival tissues from OS [296,297]. Additionally, chicory contributes to the microbiological balance of the oral cavity and may reduce inflammation, preventing periodontal diseases [382]. Its active compounds stimulate salivary secretion, offering protection against xerostomia. Studies have shown that *C. intybus* extracts exhibit antimicrobial effects on oral bacteria, inhibiting the virulence of pathogens such as *Prevotella intermedia*, *Actinomyces*, and *S. mutans*. Another study reported the use of latex extracted from *C. intybus* to facilitate the extraction of carious molars, due to its therapeutic properties [383].

These findings suggest that *Cichorium intybus* L. may have promising applications in the prevention and treatment of oral diseases, serving as a natural alternative for maintaining gingival and dental health.

#### 8.2.3. *Calendula officinalis* L.

*Calendula officinalis*, commonly known as marigold, is a medicinal plant that is recognized for its antioxidant, anti-inflammatory, and antimicrobial properties, widely used in oral care [384]. It contains flavonoids and triterpenes, which protect cells against OS and free radicals [385,386]. Quercetin from *Calendula officinalis* significantly contributes to antioxidant activity, with an optimal structure for neutralizing free radicals [387]. The beneficial effects of marigold were demonstrated in a study on experimental periodontitis in rats, where the administration of *Calendula officinalis* extract reduced OS in periodontal tissues by restoring the activity of antioxidant enzymes (GSH, SOD, CAT) and decreasing MDA levels [388,389,390]. Since inflammation and OS are major factors in the pathogenesis of periodontal disease and other oral conditions, *Calendula officinalis* can be used as an adjuvant in prevention and treatment, protecting the gums and maintaining oral health.

#### 8.2.4. *Taraxacum officinale* L.

*Taraxacum officinale* L., commonly known as dandelion, is a medicinal plant that is valued for its complex chemical composition and therapeutic properties. It contains polyphenols, flavonoids, triterpenoids, and sterols, contributing to its antioxidant and anti-inflammatory activity [391]. Although numerous studies have demonstrated its free-radical-neutralizing capacity, its specific effects on oral health remain limited. However, due to their antioxidant properties, *Taraxacum officinale* extracts may play a role in preventing and managing oral conditions, such as gingival inflammation and mucosal lesions. Further clinical studies are needed to confirm its efficacy and safety in oral health applications.

#### 8.2.5. *Arctium lappa* L.

*Arctium lappa* L. (burdock) is a perennial plant that is valued in traditional medicine for its therapeutic properties. Its roots contain phenolic compounds, such as chlorogenic acid and quercitrin, which contribute to its antioxidant activity [392]. In dentistry, *Arctium lappa* has demonstrated antimicrobial effects against oral microorganisms, including those involved in endodontic infections [393,394]. Additionally, its extracts are effective against *Candida albicans*, showing antifungal potential [395]. Due to its antioxidant properties, burdock helps maintain redox balance in the oral cavity, influencing salivary antioxidant levels and supporting oral health.

#### 8.2.6. *Achillea millefolium* L.

*Achillea millefolium* L. (yarrow) is a medicinal plant that is rich in polyphenols, flavonoids, and phenolic acids, known for its antioxidant and anti-inflammatory properties. Its essential oil contains chamazulene, a bioactive compound with therapeutic effects [396]. In dentistry, *Achillea millefolium* has shown efficacy in treating oral mucositis, a common complication of chemotherapy, due to its high flavonoid and tannin contents, which promote oral mucosa regeneration [397,398]. Thus, *Achillea millefolium* extracts may serve as adjuvants in oral care, supporting the healing of oral lesions and reducing inflammation.

#### 8.2.7. *Solidago virgaurea* L.

*Solidago virgaurea* L. (goldenrod) is a perennial plant that has traditionally been used in European medicine, known for its high contents of flavonoids, triterpenic glycosides, and triterpenoid saponins, which might provide antioxidant and anti-inflammatory properties [399]. In dentistry, *Solidago virgaurea* has proven beneficial in the prevention and management of gingival inflammation and mucosal lesions [400]. A recent study demonstrated that a toothpaste enriched with *Solidago virgaurea* extract reduced oral microbial biomass, showing effects comparable to those of synthetic antiseptics, but without negative side effects [401]. Studies have shown that the oral microbiome plays a significant role in overall health, including the prevention of respiratory infections in children. Additionally, the extract was effective against both bacteria and fungi in the oral cavity [402]. Due to its biodegradability and safety for both the host and environment, *Solidago virgaurea* represents a valuable natural alternative to synthetic antiseptics in daily oral care [401]

## 9. Future Research Directions

To advance the understanding and therapeutic application of medicinal plants in oral OS management, several research avenues warrant further exploration:

Mechanistic and molecular investigations: Elucidation of the precise molecular pathways by which phytochemical antioxidants modulate redox signaling, immune responses, and oral microbiota homeostasis is critical. Particular emphasis should be placed on their role in chronic inflammatory states such as periodontitis and oral carcinogenesis, where dysregulated redox and immune networks coexist.

Standardization and phytopharmaceutical formulation: Establishing standardized profiles for plant-derived formulations—including active compound quantification and dose optimization—is essential to ensure consistent therapeutic outcomes. The formulation of oral care products such as mouthwashes, gels, or dietary supplements containing extracts from Lamiaceae and Asteraceae species must undergo rigorous assessment for long-term safety, efficacy, and mucosal compatibility.

Clinical validation: Well-designed, randomized, placebo-controlled clinical trials are needed to substantiate the in vivo antioxidant and anti-inflammatory effects of these botanical species. Such trials should assess their impact on oral redox homeostasis, lesion resolution, and progression of oral pathologies.

Salivary biomarker profiling: The development and validation of robust salivary biomarkers reflecting OS and antioxidant defense—such as MDA, SOD, GSH, and 8-hydroxy-2′-deoxyguanosine (8-OHdG)—would enable non-invasive early diagnosis, real-time monitoring of therapeutic efficacy, and personalized intervention strategies.

Microbiota-targeted research: Future studies should investigate how bioactive compounds from medicinal plants affect the composition, metabolic activity, and resilience of the oral microbiota under OS. These insights may drive the development of novel microbiome-centered therapies aimed at restoring oral microbial homeostasis and preventing dysbiosis-associated diseases.

Interdisciplinary integration: Progress in this field will require the integration of knowledge from oral microbiology, phytotherapy, molecular biology, pharmacognosy, and clinical dentistry. Collaborative, cross-disciplinary approaches will facilitate the design of innovative treatment protocols that combine conventional oral care with evidence-based antioxidant interventions.

## 10. Conclusions

OS plays a central role in the initiation and progression of oral diseases, ranging from dental caries and periodontal disorders to precancerous lesions and oral cancer. The oral cavity is constantly exposed to both endogenous and exogenous sources of ROS, and when their production exceeds the neutralizing capacity of saliva, a redox imbalance arises, promoting inflammation, tissue degradation, and delayed healing. This review emphasizes the essential role of redox balance in maintaining oral health and demonstrates that both the oral microbiota and the host immune response are deeply involved in the oxidative–inflammatory cycle. Mitochondrial dysfunction, phagocyte activation, and oral microbiota dysbiosis significantly amplify OS, particularly in the presence of aggravating factors such as smoking, alcohol consumption, exposure to xenobiotics, and unbalanced diets. Under these conditions, the integrity of oral structures is compromised by processes such as lipid peroxidation, protein oxidation, DNA damage, and activation of pro-inflammatory enzymes and cytokines. Saliva plays a vital role in protecting the oral cavity, not only through its physiological functions—lubrication, digestion, and antimicrobial defense—but also through its antioxidant capacity, provided by enzymes and bioactive molecules. However, during episodes of intense OS, these defense mechanisms can be overwhelmed, contributing to the onset and worsening of oral pathologies. Medicinal plants from the Lamiaceae and Asteraceae families offer a complementary natural solution, being rich in polyphenols, flavonoids, terpenes, and phenolic acids. These compounds can modulate the oxidative response, support the antioxidant defense mechanisms of saliva, and protect oral structures from ROS-induced damage. The integration of these plants into oral hygiene products, or as adjuvant therapies, can significantly contribute to maintaining redox homeostasis and preventing inflammatory and degenerative complications in the oral cavity.

## Figures and Tables

**Table 1:** Plant species of the Lamiaceae family with antioxidant constituents.

Scientific Name	Plant Product Used	Antioxidant Compounds	References
*Lavandula angustifolia* L. (lavender)	*Lavandulae flores*	Phenolic, apigenin, kaempferol, caftaric acid, essential oil, anthocyanins, phytosterols, minerals, tannins, linalool, borneol, linalyl acetate, terpene compounds (oxygenated monoterpenes), sesquiterpenes, triterpenoids, coumarins, and aliphatic compounds (hydrocarbons, ketones, esters, etc.).	[219,220,221,222,223,224,225,226,227,228,229]
*Menthax piperita* L. (peppermint)	*Menthae Folium*	Flavonoids and methoxylated flavones, lipophilic tannins, polyphenolic substances, essential oils, amino acids such as L-serine, threonine, 4-aminobutyric acid, 5-oxo-L-proline, and unsaturated fatty acids.	[230,231,232,233,234,235,236,237,238]
*Ocimum* *basilicum* L. (basil)	*Basilici* *herba*	Polyphenols (phenolic acids, flavonoids), alkaloids, tannins, essential oil rich in linalool, estragole, methyl chavicol, eugenol, ocimene, anethole, and tannoids.	[239,240,241,242,243]
*Rosmarinus officinalis* L. (rosemary)	*Rosmarini folium*	Phenolic compounds: flavonoids (mainly flavones, but also flavonols and flavanones), phenolic acids, diterpenoids (carnosic acid, carnosol, and rosmanol derivatives), triterpenoid (betulinic acid), lignans (medioresinol derivatives), and volatile molecules (mainly terpenes, alcohols, esters, aldehydes, and ketones).	[244,245,246,247,248,249,250,251,252,253]
*Salvia* *officinalis* L. (sage)	*Salviae* *folium*	Phenolic compounds, essential oil, tannins (rosmarinic acid), flavonoids, and vitamins (ascorbic acid, vitamin B1).	[254,255,256,257,258]
*Satureja* *hortensis* L. (summer savory)	*Saturejae* *herba*	Polyphenols, volatile oil, triterpenic acids, tannins, mucilage, resins, mineral salts, gamma-terpinene, carvacrol, cymene, alpha-terpinene, alpha-pinene, myrcene, beta-caryophyllene, borneol, caryophyllene oxide, alpha-terpineol, thymol, camphene and camphor, nitrogenous substances, cellulose, essential oils, ursolic acid, oleanolic acid, mucilages, resins, sisterine, and vitamins B1 and C.	[259,260,261,262,263,264,265,266,267,268]
*Thymus* *serpyllum* L. (thyme)	*Serpylli* *herba*	Volatile oils (borneol, thymol, carvacrol), caffeic and rosmarinic acids, tannins and thymol, polyphenols, phenolic acids, caffeic acids, anthocyanins, flavonoids, inulin, carotenes, ascorbic acid, vitamin A, vitamin C, and vitamin E.	[255,269,270,271,272,273,274]
*Thymus* *vulgaris* L. (garden thyme)	*Thymi* *herba*	Phenolic compounds, vitamins (A, C, and E), essential oils, thymol, p-cymene, γ-terpinene, borneol, carvacrol, β-linalool, *cis*-sabinene hydrate, eucalyptol, α-terpinene, carvacrol methyl ether, and thymol methyl ether.	[275,276,277,278,279,280,281,282]

**Table 2:** Plant species of the Asteraceae family with antioxidant constituents.

Scientific Name	Plant Product Used	Antioxidant Compounds	References
*Matricaria chamomilla* L. (chamomile)	*Matricaria* *flower*	Apigenin, quercetin, luteolin, terpenoids, phenolic compounds, phenolic acids, flavonoids, coumarins, and essential oil.	[284,285,286,287,288,289,290,291]
*Cichorium intybus* L. (chicory)	*Cichorium* *flower*	Phenolic acids (chlorogenic acids, isochlorogenic acids, neochlorogenic acids, caffeic acid, and cichoric acid), flavonoids and polyphenols, and vitamins A and C.	[292,293,294,295,296,297,298,299]
*Calendula officinalis* L. (pot marigold)	*Calendulae flos cum* *receptaculis*	Carotenoids, flavonoids, polyphenols, volatile oil, saponosides, carotenoids, flavonoids, flavonoid glycosides, vitamin C, malic acid, protein substances, and esters.	[300,301,302,303,304,305,306]
*Taraxacum officinale* L. (dandelion)	*Taraxaci* *flos*	Phenolic compounds and flavonoids, hydroxycinnamic acid, cichoric acid, caftaric acid, chlorogenic acids, free aglycones and flavonoid glycosides, flavones such as luteolin, chrysoeriol, and apigenin, and various flavonols, such as quercetin and its O-glucoside and rutinoside glycosides.	[307,308,309,310,311,312]
*Arctium lappa* L. (greater burdock)	*Radix* *bardane* *Bardane* *folium*	Phenolic acids—primarily isomers of dicaffeoylquinic acid and their derivatives, caffeic acids, tannins, anthocyanins, flavonoids, polyphenols, inulin, and carotenes. Ascorbic acid, arctiin and arctigenin from the lignan group, and onopordopicrin from the sesquiterpene lactone group, as well as flavonoids—luteolin, quercetin, quercitrin, and rutin—and phenolic acids.	[313,314,315,316,317,318,319]
*Achillea millefolium* L. (yarrow)	*Millefolii* *flores* *Millefolii* *herba*	Volatile oil containing sesquiterpenes (mainly azulene and achillein lactone), tannins, flavonoids, and acids. Achilleic acid, formic acid, acetic acid, aconitic acid, ascorbic acid, folic acid, propionic acid, valerianic acid, palmitic acid, stearic acid, myristic acid, linoleic acid, onecinic acid, and flavones.	[320,321,322]
*Solidago virgaurea* L. (goldenrod)	*Solidaginis* *herba*	Volatile oil, polyphenolcarboxylic acid derivatives, quercetol, rutin, anthocyanins, flavonoids (quercetin, kaempferol), and terpenes (essential oil).	[323,324,325,326,327]

## Abbreviations

The following abbreviations are used in this manuscript:

OS	Oxidative stress
ROS	Reactive oxygen species
RNS	Reactive nitrogen species
H_2_O_2_	Hydrogen peroxide
NO	Nitric oxide
SOD	Superoxide dismutase
CAT	Catalase
GPx	Glutathione peroxidase
GSH	Glutathione
NOS	Nitric oxide synthase
ADH	Alcohol dehydrogenase
LPO	Lactoperoxidase
MPO	Myeloperoxidase
•OH	Hydroxyl radical
ROO•	Peroxyl radical
RO•	Alkoxyl radical
ONOO^−^	Peroxynitrite anion
O_2_^−^	Superoxide anion
UA	Uric acid
MMPs	Metalloproteinases
HA	Hydroxyapatite
MDA	Malondialdehyde
TAC	Total antioxidant capacity
OSSC	Oral squamous-cell carcinoma
*P. gingivalis*	*Porphyromonas gingivalis*
*S. mutans*	*Streptococcus mutans*

## References

[1] Peres M.A., Macpherson L.M.D., Weyant R.J., Daly B., Venturelli R., Mathur M.R., Listl S., Celeste R.K., Guarnizo-Herreño C.C., Kearns C. (2019). Oral diseases: A global public health challenge. Lancet.

[2] Sies H. (2015). Oxidative stress: A concept in redox biology and medicine. Redox Biol..

[3] El-Bahr S.M. (2013). Biochemistry of free radicals and oxidative stress. Sci. Int..

[4] Liguori I., Russo G., Curcio F., Bulli G., Aran L., Della-Morte D., Gargiulo G., Testa G., Cacciatore F., Bonaduce D. (2018). Oxidative stress, aging, and diseases. Clin. Interv. Aging.

[5] Snezhkina A.V., Kudryavtseva A.V., Kardymon O.L., Savvateeva M.V., Melnikova N.V., Krasnov G.S., Dmitriev A.A. (2019). ROS Generation and Antioxidant Defense Systems in Normal and Malignant Cells. Oxid. Med. Cell Longev..

[6] Bondeva T., Wolf G. (2014). Reactive oxygen species in diabetic nephropathy: Friend or foe?. Nephrol. Dial. Transplant..

[7] Dong Z., Wu L., Hong H. (2023). Mitochondrial Dysfunction in the Pathogenesis and Treatment of Oral Inflammatory Diseases. Int. J. Mol. Sci..

[8] Reddy V.P. (2023). Oxidative Stress in Health and Disease. Biomedicines.

[9] Brajdeș D.C., Bahrim G., Dinică R., Vizireanu C. (2013). Phenolics composition and their biochemical stability confirmation by in vitro, gastrointestinal conditions simulation, for a new functional fermented beverage based on sprouted buckwheat, Rom. Biotechnol. Lett..

[10] Suvarna R., Rao P.K., Poonja P.A., Rai D., Kini R., Meghana H.C. (2023). Salivary superoxide dismutase activity in smokeless tobacco consumers and non-consumers: A biochemical study. J. Cancer Res. Ther..

[11] Sculley D.V., Langley-Evans S.C. (2003). Periodontal disease is associated with lower antioxidant capacity in whole saliva and evidence of increased protein oxidation. Clin. Sci..

[12] Mafimisebi T.E., Oguntade A.E., Ajibefun I.A., Mafimisebi O.E., Ikuemonisan E.S. (2013). The Expanding Market for Herbal, Medicinal and Aromatic Plants In Nigeria and the International Scene. Med. Aromat. Plants.

[13] Anwar M.A., Sayed G.A., Hal D.M., El Hafeez M.S.A., Shatat A.-A.S., Salman A., Eisa N.M., Ramadan A., El-Shiekh R.A., Hatem S. (2025). Herbal remedies for oral and dental health: A comprehensive review of their multifaceted mechanisms including antimicrobial, anti-inflammatory, and antioxidant pathways. Inflammopharmacology.

[14] Moro L. (2019). Mitochondrial Dysfunction in Aging and Cancer. J. Clin. Med..

[15] Zhao M., Wang Y., Li L., Liu S., Wang C., Yuan Y., Yang G., Chen Y., Cheng J., Lu Y. (2021). Mitochondrial ROS promote mitochondrial dysfunction and inflammation in ischemic acute kidney injury by disrupting TFAM-mediated mtDNA maintenance. Theranostics.

[16] Rho J.H., Kim H.J., Joo J.Y., Lee J.Y., Lee J.H., Park H.R. (2021). Periodontal Pathogens Promote Foam Cell Formation by Blocking Lipid Efflux. J. Dent. Res..

[17] Xu T., Dong Q., Luo Y., Liu Y., Gao L., Pan Y., Zhang D. (2021). *Porphyromonas gingivalis* infection promotes mitochondrial dysfunction through Drp1-dependent mitochondrial fission in endothelial cells. Int. J. Oral. Sci..

[18] Vaseenon S., Weekate K., Srisuwan T., Chattipakorn N., Chattipakorn S. (2023). Observation of Inflammation, Oxidative Stress, Mitochondrial Dynamics, and Apoptosis in Dental Pulp following a Diagnosis of Irreversible Pulpitis. Eur. Endod. J..

[19] Dogan Buzoglu H., Ozcan M., Bozdemir O., Aydin Akkurt K.S., Zeybek N.D., Bayazit Y. (2023). Evaluation of oxidative stress cycle in healthy and inflamed dental pulp tissue: A laboratory investigation. Clin. Oral Investig..

[20] Vengerfeldt V., Mändar R., Saag M., Piir A., Kullisaar T. (2017). Oxidative stress in patients with endodontic pathologies. J. Pain Res..

[21] Papinska J.A., Durślewicz J., Bagavant H., Deshmukh U.S. (2024). Deleting Mitochondrial Superoxide Dismutase 2 in Salivary Gland Ductal Epithelial Cells Recapitulates Non-Sjögren’s Sicca Syndrome. Int. J. Mol. Sci..

[22] Katsiougiannis S., Stergiopoulos A., Moustaka K., Havaki S., Samiotaki M., Stamatakis G., Tenta R., Skopouli F.N. (2023). Salivary gland epithelial cell in Sjögren’s syndrome: Metabolic shift and altered mitochondrial morphology toward an innate immune cell function. J. Autoimmun..

[23] Zhao Y., Peng C., Zhang J., Lai R., Zhang X., Guo Z. (2022). Mitochondrial Displacement Loop Region SNPs Modify Sjögren’s Syndrome Development by Regulating Cytokines Expression in Female Patients. Front. Genet..

[24] De Benedittis G., Latini A., Colafrancesco S., Priori R., Perricone C., Novelli L., Borgiani P., Ciccacci C. (2022). Alteration of Mitochondrial DNA Copy Number and Increased Expression Levels of Mitochondrial Dynamics-Related Genes in Sjögren’s Syndrome. Biomedicines.

[25] Hajishengallis G., Lamont R.J. (2014). Breaking bad: Manipulation of the host response by *Porphyromonas gingivalis*. Eur. J. Immunol..

[26] Afzal S., Abdul Manap A.S., Attiq A., Albokhadaim I., Kandeel M., Alhojaily S.M. (2023). From imbalance to impairment: The central role of reactive oxygen species in oxidative stress-induced disorders and therapeutic exploration. Front. Pharmacol..

[27] Radi R. (2018). Oxygen radicals, nitric oxide, and peroxynitrite: Redox pathways in molecular medicine. Proc. Natl. Acad. Sci. USA.

[28] Saluja H.M., Sachdeva S., Mani A. (2021). Role of reactive oxygen species and antioxidants in periodontal disease. J. Cell. Biotechnol..

[29] Kilian M., Chapple I.L.C., Hannig M., Marsh P.D., Meuric V., Pedersen A.M.L., Tonetti M.S., Wade W.G., Zaura E. (2016). The oral microbiome—An update for oral healthcare professionals. Br. Dent. J..

[30] Cheng X., Xu X., Zhou X., Ning J. (2023). Oxidative stress response: A critical factor affecting the ecological competitiveness of *Streptococcus mutans*. J. Oral Microbiol..

[31] Waszkiewicz N., Szulc A., Zalewska A. (2017). Does Oxidative Stress Induced by Alcohol Consumption Affect Salivary Glands and the Composition of Saliva?. Front. Physiol..

[32] Waszkiewicz N., Jelski W., Zalewska A., Szulc A., Szmitkowski M., Zwierz K., Szajda S.D. (2014). Salivary alcohol dehydrogenase in non-smoking and smoking alcohol-dependent persons. Alcohol.

[33] Contreras-Zentella M.L., Villalobos-García D., Hernández-Muñoz R. (2022). Ethanol Metabolism in the Liver, the Induction of Oxidant Stress, and the Antioxidant Defense System. Antioxidants.

[34] Waszkiewicz N., Chojnowska S., Zalewska A., Szulc A., Zwierz K., Szajda S.D. (2013). Salivary hexosaminidase in smoking alcoholics with bad periodontal and dental states. Drug Alcohol Depend..

[35] Waszkiewicz N., Szajda S.D., Jankowska A., Kępka A., Dobryniewski J., Szulc A., Zwierz K. (2008). The effect of the binge drinking session on the activity of salivary, serum, urinary beta hexosaminidase: Preliminary data. Alcohol Alcohol..

[36] Watjen W., Beyersmann D. (2004). Cadmium-induced apoptosis in C6 glioma cells: Influence of oxidative stress. Biometals.

[37] Ryder M.I. (2007). The influence of smoking on host responses in periodontal infections. Periodontology 2000.

[38] Valko M., Rhodes C.J., Moncol J., Izakovic M., Mazur M. (2006). Free radicals, metals and antioxidants in oxidative stress-induced cancer. Chem.-Biol. Interact..

[39] Fiaschi A.I., Cozzolino A., Ruggiero G., Giorgi G. (2005). Glutathione, ascorbic acid and antioxidant enzymes in the tumor tissue and blood of patients with oral squamous cell carcinoma. Eur. Rev. Med. Pharmacol. Sci..

[40] Bain C.A., Weng D., Meltzer A., Kohles S.S., Stach R.M. (2002). A meta-analysis evaluating the risk for implant failure in patients who smoke. Compend. Contin. Educ. Dent..

[41] Vicidomini C., Palumbo R., Moccia M., Roviello G.N. (2024). Oxidative Processes and Xenobiotic Metabolism in Plants: Mechanisms of Defense and Potential Therapeutic Implications. J. Xenobiot..

[42] Mansoor S., Ali A., Kour N., Bornhorst J., AlHarbi K., Rinklebe J., Abd El Moneim D., Ahmad P., Chung Y.S. (2023). Heavy Metal Induced Oxidative Stress Mitigation and ROS Scavenging in Plants. Plants.

[43] Sule R.O., Condon L., Gomes A.V. (2022). A Common Feature of Pesticides: Oxidative Stress-The Role of Oxidative Stress in Pesticide-Induced Toxicity. Oxid. Med. Cell Longev..

[44] Vogeley C., Sondermann N.C., Woeste S., Momin A.A., Gilardino V., Hartung F., Heinen M., Maaß S.K., Mescher M., Pollet M. (2022). Unraveling the differential impact of PAHs and dioxin-like compounds on AKR1C3 reveals the EGFR extracellular domain as a critical determinant of the AHR response. Environ. Int..

[45] Gassman N.R. (2017). Induction of oxidative stress by bisphenol A and its pleiotropic effects. Environ. Mol. Mutagen..

[46] Zhao Z. (2023). Hydroxyl radical generations form the physiologically relevant Fenton-like reactions. Free. Radic. Biol. Med..

[47] Bhattacharya P.T., Misra S.R., Hussain M. (2016). Nutritional Aspects of Essential Trace Elements in Oral Health and Disease: An Extensive Review. Scientifica.

[48] Amin W., Farah A., Mahmoud A. (2016). Quantitative analysis of trace elements in sound and carious enamel of primary and permanent dentitions. Br. J. Med. Med. Res..

[49] Mavropoulos E. (2002). Studies on the mechanisms of lead immobilization by hydroxyapatite. Environ. Sci. Technol..

[50] Qamar Z. (2017). Influence of trace elements on dental enamel properties: A review. J. Pak. Med. Assoc..

[51] Dorta D.J., Leite S., DeMarco K.C., Prado I.M., Rodrigues T., Mingatto F.E., Uyemura S.A., Santos A.C., Curti C. (2003). A proposed sequence of events for cadmium-induced mitochondrial impairment. J. Inorg. Biochem..

[52] Wang Y., Fang J., Leonard S.S., Rao K.M. (2004). Cadmium inhibits the electron transfer chain and induces reactive oxygen species. Free Radic. Biol. Med..

[53] Chandrajith R., Diyabalanage S., Dissanayake C.B. (2020). Geogenic fluoride and arsenic in groundwater of Sri Lanka and its implications to community health. Groundw. Sustain. Dev..

[54] Yang H. (2024). Associations of urinary total arsenic and arsenic species and periodontitis. Int. Dent. J..

[55] Berlin M. (2020). Mercury in dental amalgam: A risk analysis. Neurotoxicology.

[56] Joy A., Quresi A. (2020). Mecury in dental amalgam, online retail and the Minamata convention on mercury. Environ. Sci. Technol..

[57] Kaneez F.S., Masood A., Tushar D. (2014). Oxidative Stress Gated by Fenton and Haber–Weiss Reactions and Its Association with Alzheimer’s Disease. Adv. Biomed. Res..

[58] Primožič J., Poljšak B., Jamnik P., Kovač V., Čanadi Jurešić G., Spalj S. (2021). Risk Assessment of Oxidative Stress Induced by Metal Ions Released from Fixed Orthodontic Appliances during Treatment and Indications for Supportive Antioxidant Therapy: A Narrative Review. Antioxidants.

[59] Tomova Z., Tomov D., Vlahova A. (2023). The impact of dental metal restorations on the oral oxidative stress level. J. Clin. Exp. Dent..

[60] Kushibiki T., Hirasawa T., Okawa S., Ishihara M. (2013). Blue laser irradiation generates intracellular reactive oxygen species in various types of cells. Photomed. Laser Surg..

[61] Jomova K., Valko M. (2011). Advances in metal-induced oxidative stress and human disease. Toxicology.

[62] George G.N., Singh S.P., Hoover J., Pickering I.J. (2009). The chemical forms of mercury in aged and fresh dental amalgam surfaces. Chem. Res. Toxicol..

[63] Huda A., Taghreed Z., Ali Y. (2012). Trace elements and oxidative strss markers in saliva of subjects with amalgam fillings. J. Bangh. Coll. Dent..

[64] Daokar S., Siddiqui S., AlJeaidi Z.A., Mustafa M., Mapari P.S., Nadeem F. (2016). Assessment of Oxidative Stress Induced by Various Restorative Materials: An In Vivo Biochemical Study. J. Int. Oral Health.

[65] Jiang S., Liu H., Li C. (2021). Dietary Regulation of Oxidative Stress in Chronic Metabolic Diseases. Foods.

[66] Żukowski P., Maciejczyk M., Waszkiel D. (2018). Sources of free radicals and oxidative stress in the oral cavity. Arch. Oral Biol..

[67] Zalewska A., Knaś M., Żendzian-Piotrowska M., Waszkiewicz N., Szulimowska J., Prokopiuk S., Car H. (2014). Antioxidant profile of salivary glands in high fat diet-induced insulin resistance rats. Oral Dis..

[68] Mejean C., Morzel M., Neyraud E., Issanchou S., Martin C., Bozonnet S. (2015). Salivary composition is associated with liking and usual nutrient intake. PLoS ONE.

[69] Westman E.C., Yancy W.S., Edman J.S., Tomlin K.F., Perkins C.E. (2002). Effect of 6-month adherence to a very low carbohydrate diet program. Am. J. Med..

[70] Zhang Y., Liu Y., Wang Y., Wang X. (2022). Origin and Fate of Acrolein in Foods. Foods.

[71] Chang S.-W., Lee S.-I., Bae W.-J., Min K.-S., Shin E.-S., Oh G.-S., Pae H.-O., Kim E.-C. (2009). Heat Stress Activates Interleukin-8 and the Antioxidant System via Nrf2 Pathways in Human Dental Pulp Cells. J. Endod..

[72] Danielsson D., Brehwens K., Halle M., Marczyk M., Sollazzo A., Polanska J., Munck-Wikland E., Wojcik A., Haghdoost S. (2016). Influence of genetic background and oxidative stress response on risk of mandibular osteoradionecrosis after radiotherapy of head and neck cancer. Head Neck.

[73] Lambade P.N., Lambade D., Goel M. (2013). Osteoradionecrosis of the mandible: A review. Oral Maxillofac. Surg..

[74] Ayala A., Muñoz M.F., Argüelles S. (2014). Lipid Peroxidation: Production, Metabolism, and Signaling Mechanisms of Malondialdehyde and 4-Hydroxy-2-Nonenal. Oxidative Med. Cell. Longev..

[75] Gaschler M.M., Stockwell B.R. (2017). Lipid peroxidation in cell death. Biochem. Biophys. Res. Commun..

[76] Ito F., Sono Y., Ito T. (2019). Measurement and Clinical Significance of Lipid Peroxidation as a Biomarker of Oxidative Stress: Oxidative Stress in Diabetes, Atherosclerosis, and Chronic Inflammation. Antioxidants.

[77] Tsai Y.T., Yeh H.Y., Chao C.T., Chiang C.K. (2021). Superoxide dismutase 2 (SOD2) in vascular calcification: A focus on vascular smooth muscle cells, calcification pathogenesis, and therapeutic strategies. Oxid. Med. Cell Longev..

[78] Kurtul N., Gökpınar E. (2012). Salivary lipid peroxidation and total sialic acid levels in smokers and smokeless tobacco users as Maraş powder. Mediat. Inflamm..

[79] Rai B., Kharb S., Jain R., Anand S.C. (2006). Salivary lipid peroxidation product malonaldehyde in various dental diseases. World J. Med. Sci..

[80] Gokul S., Patil V.S., Jailkhani R., Hallikeri K., Kattappagari K.K. (2010). Oxidant-antioxidant status in blood and tumor tissue of oral squamous cell carcinoma patients. Oral Dis..

[81] Juan C.A., Pérez de la Lastra J.M., Plou F.J., Pérez-Lebeña E. (2021). The Chemistry of Reactive Oxygen Species (ROS) Revisited: Outlining Their Role in Biological Macromolecules (DNA, Lipids and Proteins) and Induced Pathologies. Int. J. Mol. Sci..

[82] Celec P. (2017). Oxidative Stress and Antioxidants in the Diagnosis and Therapy of Periodontitis. Front. Physiol..

[83] Davies M.J. (2005). The oxidative environment and protein damage. Biochim. Biophys. Acta.

[84] Aspee A., Lissi E.A. (2000). Acid phosphatase reaction with peroxyl radicals: Inactivation mechanism and behavior of the partially modified ensemble. Arch. Biochem. Biophys..

[85] López-Alarcón C., Arenas A., Lissi E., Silva E. (2014). The role of protein-derived free radicals as intermediaries of oxidative processes. Biomol. Concepts.

[86] Houee-Levin C., Bobrowski K. (2013). The use of the methods of radiolysis to explore the mechanisms of free radical modifications in proteins. J. Proteom..

[87] Dinç G., Fentoğlu Ö., Doğru A., İlhan İ., Kırzıoğlu F.Y., Orhan H. (2018). The evaluation of salivary oxidative stress in patients with familial mediterranean fever and chronic periodontitis. J. Periodontol..

[88] Gebicki J.M., Nauser T., Domazou A., Steinmann D., Bounds P.L., Koppenol W.H. (2010). Reduction of protein radicals by GSH and ascorbate: Potential biological significance. Amino Acids.

[89] Liu C.C., Gebicki J.M. (2012). Intracellular GSH and ascorbate inhibit radical-induced protein chain peroxidation in HL-60 cells. Free Radic. Biol. Med..

[90] Ide H., Shoulkamy M.I., Nakano T., Miyamoto-Matsubara M., Salem A.M. (2011). Repair and biochemical effects of DNA-protein cross-links. Mutat. Res..

[91] Dahiya P., Kamal R., Gupta R., Bhardwaj R., Chaudhary K., Kaur S. (2013). Reactive oxygen species in periodontitis. J. Indian Soc. Periodontol..

[92] Ling M.R., Chapple I.L., Matthews J.B. (2016). Neutrophil superoxide release and plasma C-reactive protein levels pre- and post-periodontal therapy. J. Clin. Periodontol..

[93] Daisuke E., Takaaki T., Naofumi T., Toshihiro S., Tetsuji A., Reiko Y., Tatsuo Y., Tatsuo W. (2008). Mechanical stimulation of gingiva reduces plasma 8-OHdG level in rat periodontitis. Arch. Oral Biol..

[94] Chapple I.L.C., Brock G.R., Milward M.R., Ling N., Matthews J.B. (2007). Compromised GCF total antioxidant capacity in periodontitis: Cause or effect?. J. Clin. Periodontol..

[95] Kanzaki H., Wada S., Narimiya T., Yamaguchi Y., Katsumata Y., Itohiya K., Fukaya S., Miyamoto Y., Nakamura Y. (2017). Pathways that regulate ROS scavenging enzymes, and their role in defense against tissue destruction in periodontitis. Front. Physiol..

[96] Stanisic D., Obradovic R., Vujovic S., Jovanovic M., Zivkovic V. (2019). The connection of periodontal disease and diabetes mellitus: The role of matrix metalloproteinases and oxidative stress. Serbian J. Exp. Clin. Res..

[97] Souza J.A., Rossa C.J., Garlet G.P., Nogueira A.V., Cirelli J.A. (2012). Modulation of host cell signaling pathways as a therapeutic approach in periodontal disease. J. Appl. Oral. Sci..

[98] Oben K.Z., Alhakeem S.S., McKenna M.K., Brandon J.A., Mani R., Noothi S.K., Jinpeng L., Akunuru S., Dhar S.K., Singh I.P. (2017). Oxidative stress-induced JNK/AP-1 signaling is a major pathway involved in selective apoptosis of myelodysplastic syndrome cells by Withaferin-A. Oncotarget.

[99] Melino M., Hii C.S., McColl S.R., Ferrante A. (2008). The effect of the JNK inhibitor, JIP peptide, on human T lymphocyte proliferation and cytokine production. J. Immunol..

[100] Han Y., Wang X., Zhang Y., Ding Y. (2023). NLRP3 inflammasome activity and periodontal disease pathogenesis–A comprehensive review. J. Oral Microbiol..

[101] Dewhirst F.E., Chen T., Izard J., Paster B.J., Tanner A.C., Yu W.H., Lakshmanan A., Wade W.G. (2010). The human oral microbiome. J. Bacteriol..

[102] Willis J.R., González-Torres P., Pittis A.A., Bejarano L.A., Cozzuto L., Andreu-Somavilla N., Alloza-Trabado M., Valentín A., Ksiezopolska E., Company C. (2018). Citizen science charts two major “stomatotypes” in the oral microbiome of adolescents and reveals links with habits and drinking water composition. Microbiome.

[103] Willis J.R., Gabaldón T. (2020). The Human Oral Microbiome in Health and Disease: From Sequences to Ecosystems. Microorganisms.

[104] Džunková M., Martinez-Martinez D., Gardlík R., Behuliak M., Janšáková K., Jiménez N., Vázquez-Castellanos J.F., Martí J.M., D’auria G., Bandara H.M. (2018). Oxidative stress in the oral cavity is driven by individual-specific bacterial communities. Biofilms Microbiomes.

[105] Hyde A., Parisot J., McNichol A., Bonev A. (2007). Nisin-induced changes in Bacillus morphology suggest a paradigm of antibiotic action. Proc. Natl. Acad. Sci. USA.

[106] Pisano M., Giordano F., Sangiovanni G., Capuano N., Acerra A., D’Ambrosio F. (2023). The Interaction between the Oral Micro-biome and Systemic Diseases: A Narrative Review. Microbiol. Res..

[107] Rocha F.G., Moye Z.D., Ottenberg G., Tang P., Campopiano D.J., Gibson F.C., Davey M.E. (2020). *Porphyromonas gingivalis* Sphingolipid Synthesis Limits the Host Inflammatory Response. J. Dent. Res..

[108] Di Spirito F. (2022). Oral-Systemic Health and Disorders: Latest Prospects on Oral Antisepsis. Appl. Sci..

[109] Kołodziej U., Maciejczyk M., Miąsko A., Matczuk J., Knaś M., Żukowski P., Zalewska A. (2017). Oxidative modification in the salivary glands of high fat-diet induced insulin resistant rats. Front. Physiol..

[110] Gasmi B.A., Noor S., Menzel A., Gasmi A. (2021). Oral Aphthous: Pathophysiology, Clinical Aspects and Medical Treatment. Arch. Razi. Inst..

[111] Avci E., Akarslan Z.Z., Erten H., Coskun-Cevher S. (2014). Oxidative stress and cellular immunity in patients with recurrent aphthous ulcers. Braz. J. Med. Biol. Res..

[112] Ghasemi S., Farokhpour F., Mortezagholi B. (2023). Systematic review and meta-analysis of oxidative stress and antioxidant markers in recurrent aphthous stomatitis. PLoS ONE.

[113] Hernández-Ríos P., Pussinen P.J., Vernal R., Hernández M. (2017). Oxidative Stress in the Local and Systemic Events of Apical Periodontitis. Front. Physiol..

[114] Morgan M., Liu Z.G. (2011). Crosstalk of reactive oxygen species and NF-κB signaling. Cell Res..

[115] Estornut C., Rinaldi G., Carceller M.C., Estornut S., Pérez-Leal M. (2024). Systemic and local effect of oxidative stress on recurrent aphthous stomatitis: Systematic review. J. Mol. Med..

[116] Waddington R.J., Moseley R., Embery G. (2000). Periodontal disease mechanisms—Reactive oxygen species: A potential role in the pathogenesis of periodontal diseases. Oral Dis..

[117] Canakci C.F., Cicek Y., Canakci V. (2005). Reactive oxygen species and human inflammatory periodontal diseases. Biochemistry.

[118] Mancini A., Raimondo S., Persano M., Silvestrini A. (2023). Oxidative Stress and Inflammation in Osteoporosis. Antioxidants.

[119] Han Y., Huang Y., Gao P., Yang Q., Jia L., Zheng Y., Li W. (2022). Leptin aggravates periodontitis by promoting M1 polarization via NLRP3. J. Dent. Res..

[120] Liu X., Hou Y., Yang M., Xin X., Deng Y., Fu R., Xiang X., Cao N., Liu X., Yu W. (2023). N-Acetyl-L-Cysteine-derived carbonized polymer dots with ROS scavenging via keap1-nrf2 pathway regulate alveolar bone homeostasis in periodontitis. Adv. Healthc. Mater..

[121] Aas J.A., Griffen A.L., Dardis S.R., Lee A.M., Olsen I., Dewhirst F.E., Leys E.J., Paster B.J. (2008). Bacteria of dental caries in primary and permanent teeth in children and young adults. J. Clin. Microbiol..

[122] Kajfasz J.K., Rivera-Ramos I., Scott-Anne K., Gregoire S., Abranches J., Lemos J.A. (2015). Transcription of oxidative stress genes is directly activated by SpxA1 and, to a lesser extent, by SpxA2 in *Streptococcus mutans*. J. Clin..

[123] Buczko P., Zalewska A., Szarmach I. (2015). Saliva and oxidative stress in oral cavity and in some systemic disorders. J. Physiol. Pharmacol..

[124] Rahman M.T., Hossain A., Pin C.H., Yahya N.A. (2018). Zinc and metallothionein in the development and progression of dental caries. Biol. Trace. Elem. Res..

[125] de Sousa Né Y.G., Frazão D.R., Bittencourt L.O., Fagundes N.C.F., Marañón-Vásquez G., Crespo-Lopez M.E., Maia L.C., Lima R.R. (2022). Are Dental Caries Associated with Oxidative Stress in Saliva in Children and Adolescents? A Systematic Review. Metabolites.

[126] Warnakulasuriya S., Johnson N.W., van der Waal I. (2007). Nomenclature and classification of potential malignant disorders of the oral mucosa. J. Oral Pathol. Med..

[127] Petti S. (2003). Pooled estimate of world leukoplakia prevalence: A systematic review. Oral Oncol..

[128] Sardaro N., Della Vella F., Incalza M.A., Di Stasio D., Lucchese A., Contaldo M., Laudadio C., Petruzzi M. (2019). Oxidative stress and oral mucosal diseases: An overview. In Vivo.

[129] Żukowski P., Maciejczyk M., Matczuk J., Kurek K., Waszkiel D., Żendzian Piotrowska M., Zalewska A. (2018). Effect of N-acetylcysteine on antioxidant defense, oxidative modification, and salivary gland function in a rat model of insulin resistance. Oxid. Med. Cell Longev..

[130] Onopiuk B.M., Dąbrowska Z.N., Rogalska J., Brzóska M.M., Dąbrowski A., Bijowski K., Onopiuk P., Mroczko B., Orywal K., Dąbrowska E. (2021). The beneficial impact of the black chokeberry extract against oxidative stress in the sublingual salivary gland of rats intoxicated with cadmium. Oxid. Med. Cell Longev..

[131] Alnuaimi O., Mammdoh J., Al Allaf L. (2022). The Role of Selenium in Mitigating the Adverse Effect of Cyclophosphamide on the Rat Submandibular Salivary Glands. Egypt J. Vet. Sci..

[132] Ungureanu L.B., Grădinaru I., Ghiciuc C.M., Amălinei C., Gelețu G.L., Petrovici C.G., Stănescu R.Ș. (2023). Atrophy and Inflammatory Changes in Salivary Glands Induced by Oxidative Stress after Exposure to Drugs and Other Chemical Substances: A Systematic Review. Medicina.

[133] Pizzino G., Irrera N., Cucinotta M., Pallio G., Mannino F., Arcoraci V., Squadrito F., Altavilla D., Bitto A. (2017). Oxidative Stress: Harms and Benefits for Human Health. Oxid. Med. Cell Longev..

[134] Pisoschi A.M., Pop A. (2015). The role of antioxidants in the chemistry of oxidative stress: A review. Eur. J. Med. Chem..

[135] Battino M., Ferreiro M.S., Gallardo I., Newman H.N., Bullon P. (2002). The antioxidant capacity of saliva. J. Clin. Periodontol..

[136] Halliwell B., Gutteridge J.M. (2015). Free Radicals in Biology and Medicine.

[137] Perry J.J., Shin D.S., Getzoff E.D., Tainer J.A. (2010). The structural biochemistry of the superoxide dismutases. Biochim. Biophys. Acta.

[138] Kang W., Jia Z., Tang D., Zhang Z., Gao H., He K., Feng Q. (2019). *Fusobacterium nucleatum* facilitates apoptosis, ROS generation, and inflammatory cytokine production by activating AKT/MAPK and NF-kappaB signaling pathways in human gingival fibroblasts. Oxid. Med. Cell Longev..

[139] Rath-Deschner B., Nogueira A.V.B., Memmert S., Nokhbehsaim M., Cirelli J.A., Eick S., Miosge N., Kirschneck C., Kesting M., Deschner J. (2021). Regulation of anti-apoptotic SOD2 and BIRC3 in periodontal cells and tissues. Int. J. Mol. Sci..

[140] Lewandowski L., Kepinska M., Milnerowicz H. (2020). Alterations in concentration/activity of superoxide dismutases in context of obesity and selected single nucleotide polymorphisms in genes: SOD1, SOD2, SOD3. Int. J. Mol. Sci..

[141] Wei Q., Qian S., Li N.H., Hongle C., Zhao W.C. (2024). Superoxide dismutase 2 scavenges ROS to promote osteogenic differentiation of human periodontal ligament stem cells by regulating Smad3 in alveolar bone-defective rat. J. Periodontol..

[142] Ding D., Li N., Ge Y., Wu H., Yu J., Qiu W., Fang F. (2024). Current status of superoxide dismutase 2 on oral disease progression by supervision of ROS. Biomed. Pharmacother..

[143] Liu Y., Zha L., Li B., Zhang L., Yu T., Li L. (2014). Correlation between superoxide dismutase 1 and 2 polymorphisms and susceptibility to oral squamous cell carcinoma. Exp. Ther. Med..

[144] Lu Z., Liang J., He Q., Wan Q., Hou J., Lian K., Wang A. (2019). The serum biomarker chemerin promotes tumorigenesis and metastasis in oral squamous cell carcinoma. Clin. Sci..

[145] Galler K.M., Weber M., Korkmaz Y., Widbiller M., Feuerer M. (2021). Inflammatory response mechanisms of the dentine-pulp complex and the periapical tissues. Int. J. Mol. Sci..

[146] Subapriya R., Kumaraguruparan R., Ramachandran C.R., Nagini S. (2002). Oxidant-antioxidant status in patients with oral squamous cell carcinomas at different intraoral sites. Clin. Biochem..

[147] Bagul D.N. (2013). Serum levels of antioxidant in patients with oral squamous cell carcinoma: A preliminary study. IOSR J. Dent. Med. Sci..

[148] Giebułtowicz J., Wroczyński P., Samolczyk-Wanyura D. (2011). Comparison of antioxidant enzymes activity and the concentration of uric acid in the saliva of patients with oral cavity cancer, odontogenic cysts and healthy subjects. J. Oral Pathol. Med..

[149] Nandi A., Yan L.J., Jana C.K., Das N. (2019). Role of catalase in oxidative stress- and age-associated degenerative diseases. Oxid Med. Cell Longev..

[150] Stancill J.S., Corbett J.A. (2023). Hydrogen peroxide detoxification through the peroxiredoxin/thioredoxin antioxidant system: A look at the pancreatic β-cell oxidant defense. Vitam. Horm..

[151] Vlasits J., Jakopitsch C., Bernroitner M., Zamocky M., Furtmüller P.G., Obinger C. (2010). Mechanisms of catalase activity of heme peroxidases. Arch. Biochem. Biophys..

[152] Brunelli L., Yermilov J.S., Beckman C. (2001). Modulation of catalase peroxidatic and catalatic activity by nitric oxide. Free Radic. Biol. Med..

[153] Eiro N., Fraile M., González-Jubete A., González L.O., Vizoso F.J. (2022). Mesenchymal (Stem) Stromal Cells Based as New Therapeutic Alternative in Inflammatory Bowel Disease: Basic Mechanisms, Experimental and Clinical Evidence, and Challenges. Int. J. Mol. Sci..

[154] Glorieux C., Calderon P.B. (2017). Catalase, a remarkable enzyme: Targeting the oldest antioxidant enzyme to find a new cancer treatment approach. Biol. Chem..

[155] Sarıkaya E., Doğan S. (2020). Glutathione Peroxidase in Health and Diseases. Glutathione System and Oxidative Stress in Health and Disease. IntechOpen.

[156] Maciejczyk M., Zalewska A., Ładny J.R. (2019). Salivary antioxidant barrier, redox status, and oxidative damage to proteins and lipids in healthy children, adults, and the elderly. Oxid. Med. Cell Longev..

[157] Schlorke D., Flemmig J., Gau J., Furtmüller P.G., Obinger C., Arnhold J. (2016). New insights into thiocyanate oxidation by human myeloperoxidase. J. Inorg. Biochem..

[158] Magacz M., Kędziora K., Sapa J., Krzyściak W. (2019). The Significance of Lactoperoxidase System in Oral Health: Application and Efficacy in Oral Hygiene Products. Int. J. Mol. Sci..

[159] Nijakowski K., Jankowski J., Gruszczyński D., Surdacka A. (2023). Salivary Alterations of Myeloperoxidase in Patients with Systemic Diseases: A Systematic Review. Int. J. Mol. Sci..

[160] Sande López L., García-Mato E., de Coo A., Cruz R., Antequera D., Diz P., Carro E., Rivas B. (2025). Salivary Lactoferrin Levels and Polymorphisms in Down Syndrome Individuals with Periodontitis. J. Clin. Med..

[161] Valenti P., Antonini G. (2005). Lactoferrin: An important host defense against microbial and viral attack. Cell Mol. Life Sci..

[162] Jaiswal A., Madaan S., Acharya N., Kumar S., Talwar D., Dewani D. (2021). Salivary Uric Acid: A Noninvasive Wonder for Clinicians?. Cureus.

[163] Muchandi S., Walimbe H., Bijle M., Nankar M., Chaturvedi S., Karekar P. (2015). Comparative evaluation and correlation of salivary total antioxidant capacity and salivary pH in caries-free and severe early childhood caries children. J. Contemp. Dent. Pract..

[164] Vernerová A., Kujovská Krčmová L., Melichar B., Švec F. (2020). Non-invasive determination of uric acid in human saliva in the diagnosis of serious disorders. Clin. Chem. Lab. Med..

[165] Narang D., Jain A. (2019). Estimation of salivary uric acid in dental caries: A biochemical study. J. Adv. Med. Dent. Scie. Res..

[166] Lushchak V.I. (2001). Oxidative stress and mechanisms of protection against it in bacteria. Biochemistry.

[167] Valko M., Leibfritz D., Moncol J., Cronin M.T., Mazur M., Telser J. (2007). Free radicals and antioxidants in normal physiological functions and human disease. Int. J. Biochem. Cell Biol..

[168] Vijayavel K., Gopalakrishnan S., Thilagam H., Balasubramanian M.P. (2006). Dietary ascorbic acid and α-tocopherol mitigates oxidative stress induced by copper in the thornfish Terapon jarbua. Sci. Total Environ..

[169] Deponte M. (2013). Glutathione catalysis and the reaction mechanisms of glutathione-dependent enzymes. Biochim. Biophys. Acta..

[170] Tsai C.C., Chen H.S., Chen S.L., Ho Y.P., Ho K.Y., Wu Y.M., Hung C.C. (2005). Lipid peroxidation: A possible role in the induction and progression of chronic periodontitis. J. Periodont. Res..

[171] Dalai C., Ignat-Romanul I., Rosca E., Muresan M., Micle O., Bodog F. (2013). Correlation between histopathological aspects of periodontitis and biochemical changes of oxidative stress. Rom. J. Morphol. Embryol..

[172] Oztürk L.K.L., Furuncuoglu H., Atala M.H., Uluköylü O., Akyüz S., Yarat A. (2008). Association between dental-oral health in young adults and salivary glutathione, lipid peroxidation and sialic acid levels and carbonic anhydrase activity. Braz. J. Med. Biol. Res..

[173] De Araujo F.F., Marcon R.M., Cristante A.F., Filho T. (2024). Glutathione effect on functional and histological recovery after spinal cord injury in rats. Clinics.

[174] Kükürt A., Gelen V. (2024). Understanding Vitamin C: Comprehensive Examination of Its Biological Significance and Antioxidant Properties. Ascorbic Acid—Biochemistry and Functions.

[175] Sen Gupta P., Karmakar S., Biswas I., Ghosal J., Banerjee A., Roy S., Mandal D.P., Bhattacharjee S. (2024). Vitamin E alleviates chlorpyrifos induced glutathione depletion, lipid peroxidation and iron accumulation to inhibit ferroptosis in hepatocytes and mitigate toxicity in zebrafish. Chemosphere.

[176] Abe C., Miyazawa T., Miyazawa T. (2022). Current Use of Fenton Reaction in Drugs and Food. Molecules.

[177] Dumitru C., Dinică R.M., Bahrim G.E., Vizireanu C., Baroiu L., Iancu A.V., Drăgănescu M. (2021). New Insights into the Antioxidant Compounds of Achenes and Sprouted Buckwheat Cultivated in the Republic of Moldova. Appl. Sci..

[178] Rayman M.P. (2012). Selenium and human health. Lancet.

[179] Mortensen A., Skibsted L.H., Truscott T.G. (2001). The interaction of dietary carotenoids with radical species. Arch. Biochem. Biophys..

[180] El-Agamey A., Lowe G.M., McGarvey D.J., Mortensen A., Phillip D.M., Truscott T.G. (2004). Carotenoid radical chemistry and antioxidant/pro-oxidant properties. Arch. Biochem. Biophys..

[181] Upasana B., Jyotirekha G.H., Atta-ur-Rahman (2022). Chapter 6—Plant polyphenols as potent antioxidants: Highlighting the mechanism of antioxidant activity and synthesis/development of some polyphenol conjugates. Studies in Natural Products Chemistry.

[182] Lupoae M., Lupoae P., Borda D., Cristea V., Bocioc E. (2016). Allelopathic potential of the Ranunculus rionii L. and Ceratophyllum demersum L. extracts against microbial and microalgal cultures. Environ. Eng. Manag..

[183] Eseberri I., Trepiana J., Léniz A., Gómez-García I., Carr-Ugarte H., González M., Portillo M.P. (2022). Variability in the Beneficial Effects of Phenolic Compounds: A Review. Nutrients.

[184] Grosso G., Godos J., Currenti W., Micek A., Falzone L., Libra M., Giampieri F., Forbes-Hernández T.Y., Quiles J.L., Battino M. (2022). The Effect of Dietary Polyphenols on Vascular Health and Hypertension: Current Evidence and Mechanisms of Action. Nutrients.

[185] Durazzo A., Lucarini M., Souto E.B., Cicala C., Caiazzo E., Izzo A.A., Novellino E., Santini A. (2019). Polyphenols: A concise overview on the chemistry, occurrence, and human health. Phytother. Res..

[186] Zhang H., Tsao R. (2016). Dietary polyphenols, oxidative stress and antioxidant and anti-inflammatory effects. Curr. Opin. Food Sci..

[187] Krawczyk M., Burzynska-Pedziwiatr I., Wozniak L.A., Bukowiecka-Matusiak M. (2023). Impact of Polyphenols on Inflammatory and Oxidative Stress Factors in Diabetes Mellitus: Nutritional Antioxidants and Their Application in Improving Antidiabetic Therapy. Biomolecules.

[188] Ozgová S., Hermánek J., Gut I. (2003). Different antioxidant effects of polyphenols on lipid peroxidation and hydroxyl radicals in the NADPH-, Fe-ascorbate- and Fe-microsomal systems. Biochem. Pharmacol..

[189] Soares S., Brandão E., Guerreiro C., Soares S., Mateus N., de Freitas V. (2020). Tannins in food: Insights into the molecular perception of astringency and bitter taste. Molecules.

[190] Edo G.I., Nwachukwu S.C., Ali A.B.M., Yousif E., Jikah A.N., Zainulabdeen K., Ekokotu H.A., Isoje E.F., Igbuku U.A., Opiti R.A. (2025). A review on the composition, extraction and applications of phenolic compounds. Ecol. Front..

[191] Rogozinska M., Biesaga M. (2020). Decomposition of Flavonols in the Presence of Saliva. Appl. Sci..

[192] Schestakow A., Meyer-Probst C.T., Hannig C., Hannig M. (2023). Prevention of dental biofilm formation with polyphenols: A systematic review. Planta Med..

[193] Guo Y., Li Z., Chen F., Chai Y. (2023). Polyphenols in Oral Health: Homeostasis Maintenance, Disease Prevention, and Therapeutic Applications. Nutrients.

[194] Kumar N., Goel N. (2019). Phenolic acids: Natural versatile molecules with promising therapeutic applications. Biotechnol. Rep..

[195] Hadidi M., Liñán-Atero R., Tarahi M., Christodoulou M.C., Aghababaei F. (2024). The Potential Health Benefits of Gallic Acid: Therapeutic and Food Applications. Antioxidants.

[196] Kiokias S., Proestos C., Oreopoulou V. (2020). Phenolic Acids of Plant Origin—A Review on Their Antioxidant Activity In Vitro (O/W Emulsion Systems) Along with Their in Vivo Health Biochemical Properties. Foods.

[197] Hasnat H., Shompa S.A., Islam M.M., Alam S., Richi F.T., Emon N.U., Ashrafi S., Ahmed N.U., Chowdhury M.N.R., Fatema N. (2024). Flavonoids: A treasure house of prospective pharmacological potentials. Heliyon.

[198] Srivastava J.K., Pandey M., Gupta S. (2009). Chamomile, a novel and selective COX-2 inhibitor with anti-inflammatory activity. Life Sci..

[199] Panche A.N., Diwan A.D., Chandra S.R. (2016). Flavonoids: An overview. J. Nutr. Sci..

[200] Vo T.T.T., Chu P.M., Tuan V.P., Te J.S.L., Lee I.T. (2020). The promising role of antioxidant phytochemicals in the prevention and treatment of periodontal disease via the inhibition of oxidative stress pathways: Updated insights. Antioxidants.

[201] Das A.K., Islam M.N., Faruk M.O., Ashaduzzaman M., Dungani R. (2020). Review on tannins: Extraction processes, applications and possibilities. S. Afr. J. Bot..

[202] Nagy M., Mučaji P., Grančai D. (2011). Pharmacognosy Biogenesis of Natural Substances.

[203] Jain P.L.B., Patel S.R., Desai M.A. (2022). Patchouli oil: An overview on extraction method, composition and biological activities. J. Essent. Oil Res..

[204] Chen X., Shang S., Yan F., Jiang H., Zhao G., Tian S., Chen R., Chen D., Dang Y. (2023). Antioxidant activities of essential oils and their major components in scavenging free radicals, inhibiting lipid oxidation and reducing cellular oxidative stress. Molecules.

[205] Amorati R., Baschieri A., Morroni G., Gambino R., Valgimigli L. (2016). Peroxyl radical reactions in water solution: A gym for proton-coupled electron-transfer theories. Food Chem..

[206] Sun Z., Wang H., Wang J., Zhou L., Yang P. (2014). Chemical composition and anti-inflammatory, cytotoxic and antioxidant activities of essential oil from leaves of *Mentha piperita* grown in China. PLoS ONE.

[207] Kivrak Ş. (2018). Essential oil composition and antioxidant activities of eight cultivars of lavender and lavandin from western Anatolia. Ind. Crop. Prod..

[208] Bolouri P., Salami R., Kouhi S., Kordi M., Lajayer B.A., Hadian J., Astatkie T. (2022). Applications of essential oils and plant extracts in different industries. Molecules.

[209] Câmara J.S., Locatelli M., Pereira J.A.M., Oliveira H., Arlorio M., Fernandes I., Perestrelo R., Freitas V., Bordiga M. (2022). Behind the scenes of anthocyanins—From the health benefits to potential applications in food, pharmaceutical and cosmetic fields. Nutrients.

[210] González-Burgos E., Gómez-Serranillos M.P. (2012). Terpene compounds in nature: A review of their potential antioxidant activity. Curr. Med. Chem..

[211] Hu J.P., Takahashi N., Yamada T. (2000). Coptidis Rhizoma inhibits growth and proteases of oral bacteria. Oral Dis..

[212] Bhambhani S., Kondhare K.R., Giri A.P. (2021). Diversity in chemical structures and biological properties of plant alkaloids. Molecules.

[213] Bekut M., Brkić S., Kladar N., Dragović G., Gavarić N., Božin B. (2017). Potential of selected Lamiaceae plants in anti(retro)viral therapy. Pharmacol. Res..

[214] Lourenço S.C., Moldão-Martins M., Alves V.D. (2019). Antioxidants of Natural Plant Origins: From Sources to Food Industry Applications. Molecules.

[215] Dias D.A., Urban S., Roessner U. (2012). A historical overview of natural products in drug discovery. Metabolites.

[216] Chaachouay N., Zidane L. (2024). Plant-Derived Natural Products: A Source for Drug Discovery and Development. Drugs Drug Candidates.

[217] Kessler A., Kalske A. (2018). Plant secondary metabolite diversity and species interactions. Annu. Rev. Ecol. Evol. Syst..

[218] Paul M., Ma J.K.-C. (2011). Plant-made pharmaceuticals: Leading products and production platforms. Biotechnol. Appl. Biochem..

[219] Caprari C., Fantasma F., Monaco P., Divino F., Iorizzi M., Ranalli G., Fasano F., Saviano G. (2023). Chemical profiles, in vitro antioxidant and antifungal activity of four different *Lavandula angustifolia* L. EOs. Molecules.

[220] Dobros N., Zawada K.D., Paradowska K. (2023). Phytochemical Profiling, Antioxidant and Anti-Inflammatory Activity of Plants Belonging to the Lavandula Genus. Molecules.

[221] Nedeltcheva-Antonova D., Gechovska K., Bozhanov S., Antonov L. (2022). Exploring the Chemical Composition of Bulgarian Lavender Absolute (*Lavandula angustifolia* Mill.) by GC/MS and GC-FID. Plants.

[222] Turrini F., Beruto M., Mela L., Curir P., Triglia G., Boggia R., Zunin P., Monroy F. (2021). Ultrasound-Assisted Extraction of Lavender (*Lavandula angustifolia* Miller, Cultivar Rosa) Solid By-Products Remaining after the Distillation of the Essential Oil. Appl. Sci..

[223] Tyśkiewicz K., Konkol M., Rój E. (2019). Supercritical Carbon Dioxide (scCO_2_) Extraction of Phenolic Compounds from Lavender (*Lavandula angustifolia*) Flowers: A Box-Behnken Experimental Optimization. Molecules.

[224] Adaszyńska-Skwirzyńska M., Dzięcioł M. (2017). Comparison of phenolic acids and flavonoids contents in various cultivars and parts of common lavender (*Lavandula angustifolia*) derived from Poland. Nat. Prod. Res..

[225] Prusinowska R., Śmigielski K.B. (2014). Composition, biological properties and therapeutic effects of lavender (*Lavandula angustifolia* L.). A review. Herba Pol..

[226] Da Silva G.L., Luft C., Lunardelli A., Amaral R.H., Melo D.A.D.S., Donadio M., Nunes F.B., De Azambuja M.S., Santana J.C., Moraes C.M. (2015). Antioxidant, analgesic and anti-inflammatory effects of lavender essential oil. An. Acad. Bras. Ciências.

[227] Yadikar N., Bobakulov K., Li G., Aisa H.A. (2018). Seven new phenolic compounds from *Lavandula angustifolia*. Phytochem. Lett..

[228] Héral B., Stierlin É., Fernandez X., Michel T. (2021). Phytochemicals from the genus Lavandula: A review. Phytochem. Rev..

[229] Dong G., Bai X., Aimila A., Aisa H., Maiwulanjiang M. (2020). Study on Lavender Essential Oil Chemical Compositions by GC-MS and Improved pGC. Molecules.

[230] Dorman H.J.D., Koşar M., Başer K.H.C., Hiltunen R. (2009). Phenolic Profile and Antioxidant Evaluation of *Mentha* x *piperita* L. (Peppermint) Extracts. Nat. Prod. Commun..

[231] Ashrafi B., Rashidipour M., Marzban A., Soroush S., Azadpour M., Delfani S., Ramak P. (2019). *Mentha piperita* essential oils loaded in a chitosan nanogel with inhibitory effect on biofilm formation against *S. mutans* on the dental surface. Carbohydr. Polym..

[232] Gonçalves R.S., Battistin A., Pauletti G., Rota L., Serafini L.A. (2009). Antioxidant properties of essential oils from Mentha species evidenced by electrochemical methods. Rev. Bras. Plantas Med..

[233] Li Y., Liu Y., Ma A., Bao Y., Wang M., Sun Z. (2017). In vitro antiviral, anti-inflammatory, and antioxidant activities of the ethanol extract of *Mentha piperita* L.. Food Sci. Biotechnol..

[234] Taylan O., Cebi N., Sagdic O. (2021). Rapid screening of *Mentha spicata* essential oil and L-menthol in *Mentha piperita* essential oil by ATR-ftir spectroscopy coupled with multivariate analyses. Foods.

[235] Mahendran G., Rahman L. (2020). Ethnomedicinal, phytochemical and pharmacological updates on peppermint (*Mentha piperita*)—A review. Phytother. Res..

[236] Abdi G., Shokrpour M., Karami L., Salami S.A. (2018). Prolonged Water Deficit Stress and Methyl Jasmonate-Mediated Changes in Metabolite Profile, Flavonoid Concentrations and Antioxidant Activity in Peppermint (*Mentha × piperita* L.). Not. Bot. Horti Agrobot. Cluj-Napoca.

[237] Arrahmouni R., Ouazzani C., Er-Ramly A., Moustaghfir A., Dami A., Ballouch L. (2023). Chemical composition of Moroccan commercial essential oils of mint: *Mentha spicata*, *Mentha piperita*, and *Mentha pulegium*. Trop. J. Nat. Prod. Res..

[238] Al-Mijalli S.H., Elsharkawy E.R., Abdallah E.M., Hamed M., El Omari N., Mahmud S., Alshahrani M.M., Mrabti H.N., Bouyahya A. (2022). Determination of volatile compounds of *Mentha piperita* and *Lavandula multifida* and investigation of their antibacterial, antioxidant, and antidiabetic properties. Evid. Based Complement. Altern. Med..

[239] Romano R., De Luca L., Aiello A., Pagano R., Di Pierro P., Pizzolongo F., Masi P. (2022). Basil (*Ocimum basilicum* L.) Leaves as a Source of Bioactive Compounds. Foods.

[240] Nadeem H.R., Akhtar S., Sestili P., Ismail T., Neugart S., Qamar M., Esatbeyoglu T. (2022). Toxicity, Antioxidant Activity, and Phytochemicals of Basil (*Ocimum basilicum* L.) Leaves Cultivated in Southern Punjab, Pakistan. Foods.

[241] Zahran E.M., Abdelmohsen U.R., Khalil H.E., Desoukey S.Y., Fouad M.A., Kamel M.S. (2020). Diversitatea, potențialul fitochimic și medicinal al genului *Ocimum* L. (Lamiaceae). Phytochem. Rev..

[242] Hosamane M., Acharya A.B., Vij C., Trivedi D., Setty S.B., Thakur S.L. (2014). Evaluation of holy basil mouthwash as an adjunctive plaque control agent in a four-day plaque regrowth model. J. Clin. Exp. Dent..

[243] Hussain A.I., Anwar F., Sherazi S.T.H., Przybylski R. (2008). Chemical composition, anti-oxidant and antimicrobial activities of basil (*Ocimum basilicum*) essential oils depends on seasonal variations. Food Chem..

[244] Benyoucef F., Dib M.E.A., Arrar Z., Costa J., Muselli A. (2018). Synergistic Antioxidant Activity and Chemical Composition of Essential Oils from *Thymus fontanesii*, *Artemisia herba-alba* and *Rosmarinus officinalis*. J. Appl. Biotechnol. Rep..

[245] Cedeño-Pinos C., Martínez-Tomé M., Murcia M.A., Jordán M.J., Bañón S. (2020). Assessment of Rosemary (*Rosmarinus officinalis* L.) Extract as Antioxidant in Jelly Candies Made with Fructan Fibres and Stevia. Antioxidants.

[246] Mena P., Cirlini M., Tassotti M., Herrlinger K.A., Dall’Asta C., Del Rio D. (2016). Phytochemical Profiling of Flavonoids, Phenolic Acids, Terpenoids, and Volatile Fraction of a Rosemary (*Rosmarinus officinalis* L.) Extract. Molecules.

[247] Vallverdú-Queralt A., Regueiro J., Martínez-Huélamo M., Rinaldi Alvarenga J.F., Leal L.N., Lamuela-Raventos R.M. (2014). A comprehensive study on the phenolic profile of widely used culinary herbs and spices: Rosemary, thyme, oregano, cinnamon, cumin and bay. Food Chem..

[248] Nieto G., Ros G., Castillo J. (2018). Antioxidant and Antimicrobial Properties of Rosemary (*Rosmarinus officinalis*, L.): A Review. Medicines.

[249] Kontogianni V.G., Tomic G., Nikolic I., Nerantzaki A., Sayyad A., Stosic-Grujicic N., Stojanovic S., Gerothanassis I.P., Tzakos A.G. (2013). Phytochemical profile of *Rosmarinus officinalis* and *Salvia officinalis* extracts and correlation to their antioxidant and anti-proliferative activity. Food Chem..

[250] Bozin B., Mimica-Dukic N., Samojlik I., Jovin E. (2007). Antimicrobial and Antioxidant properties of Rosemary and Sage (*Rosmarinus officinalis* L. and *Salvia officinalis* L., Laminaceae) essential oils. J. Agric. Food Chem..

[251] Park J., Seo J.W., Ham D.Y., Choi H.J., Kim M.J., Na J.K., Kim S.K., Seong E.S. (2025). Antioxidant Activity and Phenolic Compound of Rosemary Under Artificial LED Lights. Agronomy.

[252] Bejenaru L.E., Segneanu A.-E., Bejenaru C., Biţă A., Tuţulescu F., Radu A., Ciocîlteu M.V., Mogoşanu G.D. (2025). Seasonal Variations in Chemical Composition and Antibacterial and Antioxidant Activities of *Rosmarinus officinalis* L. Essential Oil from Southwestern Romania. Appl. Sci..

[253] Chatterjee K., Tamta B., Mukopadayay S. (2022). A review on “pharmacological, phytochemical, and medicinal properties of *Rosmarinus officinalis* (Rosemary)”. Int. J. Health Sci..

[254] Dent M., Kovačević D.B., Bosiljkov T., Dragović-Uzelac V. (2017). Polyphenolic Composition and Antioxidant Capacity of Indigenous Wild Dalmatian Sage (*Salvia officinalis* L.). Croat. Chem. Acta.

[255] Maleš I., Dragović-Uzelac V., Jerković I., Zorić Z., Pedisić S., Repajić M., Garofulić I.E., Dobrinčić A. (2022). Non-Volatile and Volatile Bioactives of *Salvia officinalis* L., *Thymus serpyllum* L. and *Laurus nobilis* L. Extracts with Potential Use in the Development of Functional Beverages. Antioxidants.

[256] Pavić V., Jakovljević M., Molnar M., Jokić S. (2019). Extraction of Carnosic Acid and Carnosol from Sage (*Salvia officinalis* L.) Leaves by Supercritical Fluid Extraction and Their Antioxidant and Antibacterial Activity. Plants.

[257] Francik S., Francik R., Sadowska U., Bystrowska B., Zawiślak A., Knapczyk A., Nzeyimana A. (2020). Identification of Phenolic Compounds and Determination of Antioxidant Activity in Extracts and Infusions of Salvia Leaves. Materials.

[258] Politi M., Ferrante C., Menghini L., Angelini P., Flores G.A., Muscatello B., Braca A., De Leo M. (2022). Hydrosols from *Rosmarinus officinalis*, *Salvia officinalis*, and *Cupressus sempervirens*: Phytochemical Analysis and Bioactivity Evaluation. Plants.

[259] Bojor O. (2003). Guide of Medicinal and Aromatic Plants from A to Z.

[260] Fierascu I., Dinu-Pirvu C.E., Fierascu R.C., Velescu B.S., Anuta V., Ortan A., Jinga V. (2018). Phytochemical Profile and Biological Activities of *Satureja hortensis* L.: A Review of the Last Decade. Molecules.

[261] Samadi N., Masoum S., Mehrara B., Hosseini H. (2015). Application of linear multivariate calibration techniques to identify the peaks responsible for the antioxidant activity of *Satureja hortensis* L. and *Oliveria decumbens* Vent. essential oils by gas chromatography–mass spectrometry. J. Chromatogr. B..

[262] Jafari F., Ghavidel F., Zarshenas M.M. (2016). A Critical Overview on the Pharmacological and Clinical Aspects of Popular Satureja Species. J. Acupunct. Meridian Stud..

[263] Sharifzadeh A., Khosravi A.R., Ahmadian S. (2016). Chemical composition and antifungal activity of *Satureja hortensis* L. essential oil against planktonic and biofilm growth of Candida albicans isolates from buccal lesions of HIV+ individuals. Microb. Pathogen.

[264] Mašković J.M., Jakovljević V., Živković V., Mitić M., Kurćubić L.V., Mitić J., Mašković P.Z. (2024). Optimization of Ultrasound-Assisted Extraction of Phenolics from *Satureja hortensis* L. and Antioxidant Activity: Response Surface Methodology Approach. Processes.

[265] Plánder S., Gontaru L., Blazics B., Veres K., Kéry Á., Kareth S., Simándi B. (2012). Major antioxidant constituents from *Satureja hortensis* L. extracts obtained with different solvents. Eur. J. Lipid Sci. Technol..

[266] Tepe B., Cilkiz M. (2016). Pharmacological and phytochemical overview on Satureja. Pharm. Biol..

[267] Khlebnikova D.A., Efanova E.M., Danilova N.A., Shcherbakova Y.V., Rivera Sidorova I. (2022). Flavonoid Accumulation in an Aseptic Culture of Summer Savory (*Satureja hortensis* L.). Plants.

[268] Movahhedkhah S., Rasouli B., Seidavi A., Mazzei D., Laudadio V., Tufarelli V. (2019). Summer Savory (*Satureja hortensis* L.) Extract as Natural Feed Additive in Broilers: Effects on Growth, Plasma Constituents, Immune Response, and Ileal Microflora. Animals.

[269] Raudone L., Zymone K., Raudonis R., Vainoriene R., Motiekaityte V., Janulis V. (2017). Phenological Changes in Triterpenic and Phenolic Composition of *Thymus* L. Species. Ind. Crops Prod..

[270] Sonmezdag A.S., Kelebek H., Selli S. (2016). Characterization of Aroma-Active and Phenolic Profiles of Wild Thyme (*Thymus serpyllum*) by GC-MS-Olfactometry and LC-ESI-MS/MS. J. Food Sci. Technol..

[271] Mrkonjić Ž., Rakić D., Kaplan M., Teslić N., Zeković Z., Pavlić B. (2021). Pressurized-Liquid Extraction as an Efficient Method for Valorization of *Thymus serpyllum* Herbal Dust towards Sustainable Production of Antioxidants. Molecules.

[272] Galovičová L., Borotová P., Valková V., Vukovic N.L., Vukic M., Terentjeva M., Štefániková J., Ďúranová H., Kowalczewski P.Ł., Kačániová M. (2021). *Thymus serpyllum* Essential Oil and Its Biological Activity as a Modern Food Preserver. Plants.

[273] Ruiz-Malagón A.J., Rodríguez-Sojo M.J., Hidalgo-García L., Molina-Tijeras J.A., García F., Pischel I., Romero M., Duarte J., Diez-Echave P., Rodríguez-Cabezas M.E. (2022). The Antioxidant Activity of *Thymus serpyllum* Extract Protects against the Inflammatory State and Modulates Gut Dysbiosis in Diet-Induced Obesity in Mice. Antioxidants.

[274] Pavlić B., Mrkonjić Ž., Teslić N., Kljakić A.C., Pojić M., Mandić A., Stupar A., Santos F., Duarte A.R.C., Mišan A. (2022). Natural Deep Eutectic Solvent (NADES) Extraction Improves Polyphenol Yield and Antioxidant Activity of Wild Thyme (*Thymus serpyllum* L.) Extracts. Molecules.

[275] Afonso A.F., Pereira O.R., Cardoso S.M. (2020). Health-Promoting Effects of Thymus Phenolic-Rich Extracts: Antioxidant, Anti-Inflammatory and Antitumoral Properties. Antioxidants.

[276] Taghouti M., Martins-Gomes C., Félix L.M., Schäfer J., Santos J.A., Bunzel M., Nunes F.M., Silva A.M. (2020). Polyphenol composition and biological activity of *Thymus citriodorus* and *Thymus vulgaris*: Comparison with endemic Iberian *Thymus* species. Food Chem..

[277] Vladimir-Knežević S., Blažeković B., Kindl M., Vladić J., Lower-Nedza A.D., Brantner A.H. (2014). Acetylcholinesterase Inhibitory, Antioxidant and Phytochemical Properties of Selected Medicinal Plants of the Lamiaceae Family. Molecules.

[278] Pandur E., Micalizzi G., Mondello L., Horváth A., Sipos K., Horváth G. (2022). Antioxidant and Anti-Inflammatory Effects of Thyme (*Thymus vulgaris* L.) Essential Oils Prepared at Different Plant Phenophases on Pseudomonas aeruginosa LPS-Activated THP-1 Macrophages. Antioxidants.

[279] Dobreva K., Dimov M., Valev T., Iliev I., Damyanova S., Oprea O.B., Stoyanova A. (2024). Chemical Composition and Antioxidant Activities of Three Bulgarian Garden Thyme Essential Oils. Appl. Sci..

[280] Wesolowska A., Jadczak D. (2019). Comparison of the chemical composition of essential oils isolated from two thyme (*Thymus vulgaris* L.) cultivars. Not. Bot. Horti Agrobot. Cluj-Napoca.

[281] Mancini E., Senatore F., Del Monte D., De Martino L., Grulova D., Scognamiglio M., Snoussi M., De Feo V. (2015). Studies on chemical composition, antimicrobial and antioxidant activities of five *Thymus vulgaris* L. essential oils. Molecules.

[282] Stoilova I., Bail S., Buchbauer G., Krastanov A., Stoyanova A., Schmidt E., Jirovetz L. (2008). Chemical composition, olfactory evaluation and antioxidant effects of an essential oil of *Thymus vulgaris* L. from Germany. Nat. Prod. Commun..

[283] Rolnik A., Olas B. (2021). The Plants of the Asteraceae Family as Agents in the Protection of Human Health. Int. J. Mol. Sci..

[284] El-Assri E.-M., Eloutassi N., El Barnossi A., Bakkari F., Hmamou A., Bouia A. (2021). Wild chamomile (*Matricaria recutita* L.) from the Taounate Province, Morocco: Extraction and valorisation of the antibacterial activity of its essential oils. Trop. J. Nat. Prod. Res..

[285] El-Hefny M., Abo Elgat W.A.A., Al-Huqail A.A., Ali H.M. (2019). Essential and recovery oils from *Matricaria chamomilla* flowers as environmentally friendly fungicides against four fungi isolated from cultural heritage objects. Processes.

[286] Maynard R.C., Ogundipe S.O., Ferrarezi R.S., Suh J.H., Lombardini L. (2025). Apigenin Accumulation in *Matricaria chamomilla* and *Petroselinum crispum* Produced in a Vertical Hydroponic System. HortScience.

[287] Ghoniem A.A., El-Hai K.M.A., El-Khateeb A.Y., Eldadamony N.M., Mahmoud S.F., Elsayed A. (2021). Enhancing the potentiality of Trichoderma harzianum against pythium pathogen of beans using chamomile (*Matricaria chamomilla*, L.) flower extract. Molecules.

[288] Petrulova V., Vilkova M., Kovalikova Z., Sajko M., Repcak M. (2020). Ethylene Induction of non-enzymatic metabolic antioxidants in *Matricaria chamomilla*. Molecules.

[289] Orav A., Raal A., Arak E. (2010). Content and composition of the essential oil of *Chamomilla recutita* (L.) Rauschert from some European countries. Nat. Prod. Res..

[290] Piri E., Sourestani M.M., Khaleghi E., Mottaghipisheh J., Zomborszki Z.P., Hohmann J., Csupor D. (2019). Chemo-diversity and antiradical potential of twelve *Matricaria chamomilla* L. populations from Iran: Proof of ecological effects. Molecules.

[291] Elsemelawy S.A. (2017). Antidiabetic and antioxidative activity of chamomile (*Matricaria chamomilla* L.) powder on diabetic rats. J. Stud. Searches Specif. Educ..

[292] Pouille C.L., Ouaza S., Roels E., Behra J., Tourret M., Molinié R., Fontaine J.-X., Mathiron D., Gagneul D., Taminiau B. (2022). Chicory: Understanding the effects and effects of this functional food. Nutrients.

[293] Lupanova I.A. (2022). Study of *Cichorium intybus* L. Herb extract hepatoprotective activity in vitro and in vivo. Probl. Biol. Med. Pharm. Chem..

[294] Saeed M., Abd El-Hack M.E., Alagawany M., Arain M.A., Muhammad Arif M.A., Mirza M.A., Naveed M., Chao Sun C.S., Muhammad Sarwar M.S., Maryam Sayab M.S. (2017). Plant *Cichorium intybus* (Cichory): Chemical composition, pharmacology, nutritional and health applications. J. Int. Pharmacol..

[295] Shin H., Kim J., Heo H., Lee J., Kim Y. (2024). Comparison between antioxidant activities and functional components of roasted chicory root extracts produced using different ethanol concentrations. J. Korean Soc. Food Sci. Nutr..

[296] Nwafor I.C., Shale K., Achilonu M.C. (2017). Chemical composition and nutritional benefits of chicory (*Cichorium intybus*) as an ideal complementary and/or alternative feed for animals. Sci. World J..

[297] Puhlmann M.L., de Vos W.M. (2020). Back to the roots: Review of the use of fiber-rich taproots *Cichorium intybus* L.. Adv. Nutr..

[298] Rahimullah T.G. (2019). Phytochemical and antibacterial screening of *Cichorium intybus* seeds used in traditional medicine systems of Pakistan. Int. J. Basic Med. Pharmacol..

[299] Upadhayay V.K., Pandey D., Singh S.P., Gohat T. (2021). Chicory (*Cichorium intybus*): An ethnomedicinal plant with broad-spectrum medicinal properties. Plant Secondary Metabolites for Human Health.

[300] Ashwlayan V.D., Kumar A., Verma M., Garg V.K., Gupta S. (2018). Therapeutic Potential of *Calendula officinalis*. Pharm. Pharmacol. Int. J..

[301] Chroho M., Drioiche A., Saidi S., Zair T., Bouissane L. (2021). Total phenolic and flavonoids contents and in vitro evaluation of antioxidant activity of several *Calendula officinalis* (Marigold) extracts. J. Biol. Res..

[302] Givol O., Kornhaber R., Visentin D., Cleary M., Haik J., Harats M. (2019). A systematic review of *Calendula officinalis* extract for wound healing. Wound Repair Regen..

[303] Yalgi V.S., Bhat K.G. (2020). Compare and evaluate the antibacterial efficacy of sodium hypochlorite and *Calendula officinalis* against *Streptococcus mutans* as a root canal irrigating solution: An in vivo study. J. Int. Oral Health.

[304] Gunasekaran S., Nayagam A.A., Natarajan R. (2020). Wound healing potentials of herbal ointment containing *Calendula officinalis* Linn. on the alteration of immunological markers and biochemical parameters in excision wounded animals. Clin. Phytosci..

[305] Anand U., Jacobo-Herrera N., Altemimi A., Lakhssassi N. (2019). A comprehensive review on medicinal plants as antimicrobial therapeutics: Potential avenues of biocompatible drug discovery. Metabolites.

[306] El-Sayed M.K., Hommos A.M., Kotry G.S., Labib G.S. (2021). The effect of a calendula-based topical formula versus oxidized regenerated cellulose on palatal wound healing: A randomized controlled clinical trial. Alex. Dent. J..

[307] Martinez M., Poirrier P., Chamy R., Prüfer D., Schulze-Gronover C., Jorquera L., Ruiz G. (2015). *Taraxacum officinale* and related species—An ethnopharmacological review and its potential as a commercial medicinal plant. J. Ethnopharmacol..

[308] Majewski M., Lis B., Juśkiewicz J., Ognik K., Borkowska-Sztachańska M., Jedrejek D., Stochmal A., Olas B. (2020). Phenolic fractions from dandelion leaves and petals as modulators of the lipid profile and antioxidant status in an in vivo study. Antioxidants.

[309] Jedrejek D., Lis B., Rolnik A., Stochmal A., Olas B. (2019). Comparative phytochemical, cytotoxicity, antioxidant and hemostatic studies of *Taraxacum officinale* root preparations. Food Chem. Toxicol..

[310] Xue Y., Zhang S., Du M., Zhu M.J. (2017). Dandelion extract suppresses reactive oxidative species and inflammasome in intestinal epithelial cells. J. Funct. Foods..

[311] Lis B., Jedrejek D., Rywaniak J., Soluch A., Stochmal A., Olas B. (2020). Flavonoid Preparations from *Taraxacum officinale* L. Fruits-A Phytochemical, Antioxidant and Hemostasis Studies. Molecules.

[312] Sareedenchai V., Zidorn C. (2010). Flavonoids as chemosystematic markers in the tribe Cichorieae of the Asteraceae. Biochem. Syst. Ecol..

[313] Carlotto J., Da Silva L.M., Dartora N., Maria-Ferreira D., Sabry D.D.A., Filho A.P.S., Werner M.F.d.P., Sassaki G.L., Gorin P.A.J., Iacomini M. (2015). Identification of a dicaffeoylquinic acid isomer from *Arctium lappa* with a potent anti-ulcer activity. Talanta.

[314] Carlotto J., de Souza L.M., Baggio C.H., Werner M.F.D.P., Maria-Ferreira D., Sassaki G.L., Iacomini M., Cipriani T.R. (2016). Polysaccharides from *Arctium lappa* L.: Chemical structure and biological activity. Int. J. Biol. Macromol..

[315] Yosri N., Alsharif S.M., Xiao J., Musharraf S.G., Zhao C., Saeed A., Gao R., Said N.S., Di Minno A., Daglia M. (2023). *Arctium lappa* (Burdock): Insights from ethnopharmacology potential, chemical constituents, clinical studies, pharmacological utility and nanomedicine. Biomed Pharmacother..

[316] De Almeida A.B.A., Sánchez-Hidalgo M., Martín A.R., Luiz-Ferreira A., Trigo J.R., Vilegas W., Dos Santos L.C., Souza-Brito A.R.M., De La Lastra C.A. (2013). Anti-inflammatory intestinal activity of *Arctium lappa* L. (Asteraceae) in TNBS colitis model. J. Ethnopharmacol..

[317] Ferracane R., Graziani G., Gallo M., Fogliano V., Ritieni A. (2010). Metabolic profile of the bioactive compounds of burdock (*Arctium lappa*) seeds, roots and leaves. J. Pharm. Biomed. Anal..

[318] Chan Y.S., Cheng L.N., Wu J.H., Chan E., Kwan Y.W., Lee S.M., Leung G.P., Yu P.H., Chan S.W. (2011). A review of the pharmacological effects of *Arctium lappa* (burdock). Inflammopharmacology.

[319] Jaiswal R., Kuhnert N. (2011). Identification and characterization of five new classes of chlorogenic acids in burdock (*Arctium lappa* L.) roots by liquid chromatography/tandem mass spectrometry. Food Funct..

[320] Mouhid L., Gómez de Cedrón M., Quijada-Freire A., Fernández-Marcos P.J., Reglero G., Fornari T., Ramírez de Molina A. (2019). Yarrow supercritical extract ameliorates the metabolic stress in a model of obesity induced by high-fat diet. Nutrients.

[321] Pereira J.M., Peixoto V., Teixeira A., Sousa D., Barros L., Ferreira I.C.F.R., Vasconcelos M.H. (2018). *Achillea millefolium* L. hydroethanolic extract inhibits growth of human tumor cell lines by interfering with cell cycle and inducing apoptosis. Food Chem. Toxicol..

[322] Vidović S., Vasić A., Vladić J., Jokić S., Aladić K., Gavarić A., Nastić N. (2021). Carbon dioxide supercritical fluid extracts from yarrow and rose hip herbal dust as valuable sources of aromatic and lipophilic compounds. Sustain. Chem. Pharm..

[323] Fursenco C., Calalb T., Uncu L., Dinu M., Ancuceanu R. (2020). *Solidago virgaurea* L.: A review of its ethnomedicinal uses, phytochemistry, and pharmacological activities. Biomolecules.

[324] Woźniak D., Ślusarczyk S., Domaradzki K., Dryś A., Matkowski A. (2018). Comparison of polyphenol profile and antimutagenic and antioxidant activities in two species used as sources of *Solidaginis herba*—Goldenrod. Chem. Biodivers..

[325] European Medicines Agency (2008). Assessment Report on Solidago virgaurea L., Herba.

[326] Thiem B., Wesołowska M., Skrzypczak L., Budzianowski J. (2001). Phenolic compounds in two *Solidago* L. species from in vitro culture. Acta Pol. Pharm..

[327] Dobjanschi L., Paltinean R., Vlase L., Babota M., Fritea L., Tamas M. (2018). Comparative phytochemical research of *Solidago* genus: *S. graminifolia*. Note I. Flavonoids. Acta Biol. Marisiensis.

[328] Kumar A., Sharma R., Singh D. (2024). Plant polyphenols, terpenes, and terpenoids in oral health: A comprehensive review. J. Med. Plants Res..

[329] Miastkowska M., Kantyka T., Bielecka E., Kałucka U., Kamińska M., Kucharska M., Kilanowicz A., Cudzik D., Cudzik K. (2021). Enhanced biological activity of a novel preparation of *Lavandula angustifolia* essential oil. Molecules.

[330] Radu C.M., Radu C.C., Bochiș S.A., Arbănași E.M., Lucan A.I., Murvai V.R., Zaha D.C. (2023). Revisiting the therapeutic effects of essential oils on the oral microbiome. Pharmacy.

[331] Betlej I., Andres B., Cebulak T., Kapusta I., Balawejder M., Żurek N., Jaworski S., Lange A., Kutwin M., Pisulewska E. (2024). Phytochemical composition and antimicrobial properties of new *Lavandula angustifolia* ecotypes. Molecules.

[332] Altaei D.T. (2012). Topical lavender oil for the treatment of recurrent aphthous ulceration. Am. J. Dent..

[333] Ćavar Zeljković S., Šišková J., Komzáková K., De Diego N., Kaffková K., Tarkowski P. (2021). Phenolic compounds and biological activity of selected Mentha species. Plants.

[334] Hudz N., Kobylinska L., Pokajewicz K., Horčinová Sedláčková V., Fedin R., Voloshyn M., Myskiv I., Brindza J., Wieczorek P.P., Lipok J. (2023). *Mentha piperita*: Essential oil and extracts, their biological activities, and perspectives on the development of new medicinal and cosmetic products. Molecules.

[335] Li Z., Zhang H., Wang Y., Li Y., Li Q., Zhang L. (2022). The distinctive role of menthol in pain and analgesia: Mechanisms, practices, and advances. Front. Mol. Neurosci..

[336] Kamatou G.P., Vermaak I., Viljoen A.M., Lawrence B.M. (2013). Menthol: A simple monoterpene with remarkable biological properties. Phytochemistry.

[337] Rani N., Singla R.K., Narwal S., Tanushree, Kumar N., Rahman M.M. (2022). Medicinal plants used as an alternative to treat gingivitis and periodontitis. Evid. Based Complement. Alternat. Med..

[338] da Silva Moura E.d.S., D’Antonino Faroni L.R., Fernandes Heleno F.F., Aparecida Zinato Rodrigues A.A.Z., Figueiredo Prates L.H., Lopes Ribeiro de Queiroz M.E. (2020). Optimal extraction of *Ocimum basilicum* essential oil by association of ultrasound and hydro-distillation and its potential as a biopesticide against a major stored grains pest. Molecules.

[339] Şahin H., Turumtay E.A., Yildiz O., Kolayli S. (2015). Grayanotoxin-III detection and anti-oxidant activity of mad honey. Int. J. Food Prop..

[340] Mohamed F.H., Abd Elaziz N.Y. (2020). Impact of *Ocimum basilicum* leaves powder on immune response of chicken vaccinated against Newcastle disease virus. Egypt. J. Agric. Res..

[341] Tewari D., Pandey H.K., Sah A.N., Meena H., Chander V., Singh R., Singh P. (2015). Phytochemical, anti-oxidant and antidepressant evaluation of *Ocimum basilicum* enuiflorum kilimandscharicum grown in India. J. Biol. Active Prod. Nat..

[342] Güez C.M., Souza R.O.d., Fischer P., Leão M.F.d.M., Duarte J.A., Boligon A.A., Athayde M.L., Zuravski L., Oliveira L.F.S.D., Machado M.M. (2017). Evaluation of basil extract (*Ocimum basilicum*) on oxidative, antigenotoxic and anti-inflammatory effects in human leukocytes cell cultures exposed to challenging agents. Brazil J. Pharm. Sci..

[343] Refaey M.S., Abosalem E.F., Yasser El-Basyouni R., Elsheriri S.E., Elbehary S.H., Fayed M.A.A. (2024). Exploring the therapeutic potential of medicinal plants and their active principles in dental care: A comprehensive review. Heliyon.

[344] Andrade J.M., Faustino C., Garcia C., Ladeiras D., Reis C.P., Rijo P. (2018). *Rosmarinus officinalis* L.: An update review of its phytochemistry and biological activity. Future Sci. OA.

[345] de Oliveira J.R., de Jesus D., de Oliveira L.D. (2017). *Rosmarinus officinalis* L. (rosemary) extract decreases the biofilms viability of oral health interest. Braz. Dent. Sci..

[346] Günther M., Karygianni L., Argyropoulou A., Anderson A.C., Hellwig E., Skaltsounis A.L., Wittmer A., Vach K., Al-Ahmad A. (2022). The antimicrobial effect of *Rosmarinus officinalis* extracts on oral initial adhesion ex vivo. Clin. Oral Investig..

[347] Larsen T., Fiehn N.-E. (2017). Dental biofilm infections—An update. APMIS.

[348] Okasha M.I., Mostafa M.H., El-Araby S.M. (2022). Evaluation of antibacterial effect of *Rosmarinus officinalis* extract on *Streptococcus mutans* in children. Al-Azhar Dent. J. Girls.

[349] Valones M.A.A., Silva I.C.G., Gueiros L.A.M., Leao J.C., Caldas A.F., Carvalho A.A.T. (2019). Clinical assessment of rosemary-based toothpaste (*Rosmarinus officinalis* Linn.): A randomized controlled double-blind study. Braz. Dent. J..

[350] Zhang T., Liu C., Ma S., Gao Y., Wang R. (2020). Protective Effect and Mechanism of Action of Rosmarinic Acid on Radiation-Induced Parotid Gland Injury in Rats. Dose Response.

[351] Iacopetta D., Ceramella J., Scumaci D., Catalano A., Sinicropi M.S., Tundis R., Alcaro S., Borges F. (2023). An update on recent studies focusing on the antioxidant properties of Salvia species. Antioxidants.

[352] Ghorbani A., Esmaeilizadeh M. (2017). Pharmacological properties of *Salvia officinalis* and its components. J. Tradit. Complement. Med..

[353] Beheshti-Rouy M., Azarsina M., Rezaie-Soufi L., Alikhani M.Y., Roshanaie G., Komaki S. (2015). The antibacterial effect of sage extract (*Salvia officinalis*) mouthwash against *Streptococcus mutans* in dental plaque: A randomized clinical trial. Iran J. Microbiol..

[354] Sookto T., Srithavaj T., Thaweboon S., Thaweboon B., Shrestha B. (2013). In vitro effects of *Salvia officinalis* L. essential oil on *Candida albicans*. Asian Pac. J. Trop. Biomed..

[355] Santos T.d.S.A., Meccatti V.M., Pereira T.C., Marcucci M.C., Hasna A.A., Valera M.C., de Oliveira L.D., Carvalho C.A.T. (2023). Antibacterial effect of combinations of *Salvia officinalis* and *Glycyrrhiza glabra*, hydroalcoholic extracts against *Enterococcus* spp.. Coatings.

[356] Hagh L.G., Arefian A., Farajzade A., Dibazar S., Samiea N. (2019). The antibacterial activity of *Satureja hortensis* extract and essential oil against oral bacteria. Dent. Res. J..

[357] Mohtashami S., Rowshan V., Tabrizi L., Babalar M., Ghani A. (2018). Summer savory (*Satureja hortensis* L.) essential oil constituent oscillation at different storage conditions. Ind. Crops Prod..

[358] Ejaz A., Waliat S., Arshad M.S., Khalid W., Khalid M.Z., Rasul Suleria H.A., Luca M.I., Mironeasa C., Batariuc A., Ungureanu-Iuga M. (2023). A comprehensive review of summer savory (*Satureja hortensis* L.): Promising ingredient for production of functional foods. Front Pharmacol..

[359] Adiguzel A., Ozer H., Kilic H., Cetin B. (2007). Screening of antimicrobial activity of essential oil and methanol extract of *Satureja hortensis* on foodborne bacteria and fungi. Czech J. Food Sci..

[360] Sabzghabaee A.M., Davoodi N., Ebadian B., Aslani A., Ghannadi A. (2012). Clinical evaluation of the essential oil of *Satureja hortensis* for the treatment of denture stomatitis. Dent. Res. J..

[361] Jalil B., Pischel I., Feistel B., Suarez C., Blainski A., Spreemann R., Roth-Ehrang R., Heinrich M. (2024). Wild thyme (*Thymus serpyllum* L.): A review of the current evidence of nutritional and preventive health benefits. Front. Nutr..

[362] Jarić S., Mitrović M., Pavlović P. (2015). Review of ethnobotanical, phytochemical, and pharmacological study of *Thymus serpyllum* L.. Evid. Based Complement. Alternat. Med..

[363] Jovanović A.A., Balanč B., Petrović P., Pravilović R., Djordjević V. (2021). Pharmacological potential of *Thymus serpyllum* L. (wild thyme) extracts and essential oil: A review. J. Eng. Proces Manag..

[364] Fani M., Kohanteb J. (2017). In vitro antimicrobial activity of *Thymus vulgaris* essential oil against major oral pathogens. J. Evid. Based Complement. Altern. Med..

[365] Altındal D., Deveci K.C., Öner Talmaç A.G., Talmaç A.C., Çalışır M. (2023). Effects of thyme on halitosis in gingivitis patients: Can thyme mouthwash prevent halitosis—A randomized trial. Int. J. Dent. Hyg..

[366] Kameri A., Dragidella A., Haziri A., Hashani Z., Kurteshi K., Kurti A. (2024). Antifungal and genotoxic effects of *Thymus serpyllum* as a root canal irrigant. Clin. Exp. Dent. Res..

[367] Dasila K., Singh M. (2022). Bioactive compounds and biological activities of *Elaegnus latifolia* L.: An underutilized fruit of North-East Himalaya, India. S. Afr. J. Bot..

[368] Xiang Z., Guan H., Zhao X., Xie Q., Xie Z., Cai F., Dang R., Li M., Wang C. (2024). Dietary gallic acid as an antioxidant: A review of its food industry applications, health benefits, bioavailability, nano-delivery systems, and drug interactions. Food Res. Int..

[369] Salehi B., Prakash Mishra A., Shukla I., Sharifi-Rad M., del Mar Contreras M., Segura-Carretero A., Fathi H., Nasri Nasrabadi N., Kobarfard F., Sharifi-Rad J. (2018). Thymol, thyme, and other plant sources: Health and potential uses. Phytother. Res..

[370] Soto-Mendívil E.A., Moreno-Rodríguez J.F., Estarrón-Espinosa M., García-Fajardo J.A., Obledo-Vázquez E.N. (2006). Chemical composition and fungicidal activity of the essential oil *Thymus vulgaris* against *Alternaria citri*. e-Gnosis.

[371] Borugă O., Jianu C., Mişcă C., Goleţ I., Gruia A., Horhat F. (2014). *Thymus vulgaris* essential oil: Chemical composition and antimicrobial activity. J. Med. Life.

[372] Sas I. (2019). *Thymus vulgaris* extract formulated as cyclodextrin complexes: Synthesis, characterization, antioxidant activity and in vitro cytotoxicity assessment. Farmacia.

[373] Botelho M., Nogueira N.A.P., Bastos G., Fonseca S., Lemos T., Matos F., Montenegro D., Heukelbach J., Rao V.S., Brito G. (2007). Antimicrobial activity of the essential oil from Lippia sidoides, carvacrol and thymol against oral pathogens. Braz. J. Med. Biol. Res..

[374] Waheed M., Hussain M.B., Saeed F., Afzaal M., Ahmed A., Irfan R., Akram N., Ahmed F., Hailu G.G. (2024). Phytochemical Profiling and Therapeutic Potential of Thyme (*Thymus* spp.): A Medicinal Herb. Food Sci. Nutr..

[375] Djeridane A., Yousfi M., Nadjemi B., Boutassouna D., Stocker P., Vidal N. (2006). Antioxidant activity of some Algerian medicinal plant extracts containing phenolic compounds. Food Chem..

[376] Salehi B., Lopez-Jornet P., López E.P.-F., Calina D., Sharifi-Rad M., Ramírez-Alarcón K., Forman K., Fernández M., Martorell M., Setzer W.N. (2019). Plant-Derived Bioactives in Oral Mucosal Lesions: A Key Emphasis to Curcumin, Lycopene, Chamomile, Aloe vera, Green Tea and Coffee Properties. Biomolecules.

[377] Cai Y., Luo Q., Sun M., Corke H. (2004). Antioxidant activity and phenolic compounds of 112 transitional Chinese medicinal plants associated with anticancer. Life Sci..

[378] Chirinos R., Pedreschi R., Rogez H., Larondelle Y., Campos D. (2013). Phenolic compound contents and antioxidant activity in plants with nutritional and/or medicinal properties from the Peruvian Andean region. Ind. Crop. Prod..

[379] de Lucena R.N., Lins R.D., Ramos I.N., Cavalcanti A.L., Gomes R.C., Maciel M.D. (2009). Estudo clínico comparativo do efeito anti-inflamatório da *Matricaria recutita* e da clorexidina em pacientes com gengivite crônica. Braz. J. Med. Biol. Res..

[380] Singh Y.P., Sachdeva O.P., Aggarwal S.K., Chugh K., Lal H. (2008). Blood glutathione levels in head and neck malignancies. Indian J. Clin. Biochem..

[381] Malcangi G., Patano A., Ciocia A.M., Netti A., Viapiano F., Palumbo I., Trilli I., Guglielmo M., Inchingolo A.D., Dipalma G. (2023). Benefits of natural antioxidants on oral health. Antioxidants.

[382] Pytko-Polończyk J., Stawarz-Janeczek M., Kryczyk-Poprawa A., Muszyńska B. (2021). Antioxidant-rich natural raw materials in the prevention and treatment of selected oral cavity and periodontal diseases. Antioxidants.

[383] Street R.A., Sidana J., Prinsloo G. (2013). *Cichorium intybus*: Traditional uses, phytochemistry, pharmacology, and toxicology. Evid. Based Complement. Altern. Med..

[384] Vella F.M., Pignone D., Laratta B. (2024). The Mediterranean species *Calendula officinalis* and *Foeniculum vulgare* as valuable sources of bioactive compounds. Molecules.

[385] Ak G., Zengin G., Sinan K.I., Mahomoodally M.F., Picot-Allain M.C.N., Cakır O., Bensari S., Yılmaz M.A., Gallo M., Montesano D. (2020). A comparative bio-evaluation and chemical profiles of *Calendula officinalis* L. extracts prepared via different extraction techniques. Appl. Sci..

[386] Rigane G., Younes S.B., Ghazghazi H., Salem R.B. (2013). Investigation into the biological activities and chemical composition of *Calendula officinalis* L. growing in Tunisia. Int. Food Res. J..

[387] Heijnen C.G., Haenen G.R., Oostveen R.M., Stalpers E.M., Bast A. (2002). Protection of flavonoids against lipid peroxidation: The structure activity relationship revisited. Free Radic. Res..

[388] Fonseca Y.M., Catini C.D., Vicentini F.T., Nomizo A., Gerlach R.F., Fonseca M.J. (2010). Protective effect of *Calendula officinalis* extract against UVB-induced oxidative stress in skin: Evaluation of reduced glutathione levels and matrix metallo-proteinase secretion. J. Ethnopharmacol..

[389] Asadi-Yousefabad S.-L., Tanideh N., Jamshidzadeh A., Sepehrimanesh M., Hosseinzadeh M., Koohi-Hosseinabadi O., Najibi A., Raam M., Daneshi S. (2016). Healing acceleration of acetic acid-induced colitis by marigold (*Calendula officinalis*) in male rats. Saudi J. Gastroenterol..

[390] Verma P.K., Raina R., Sultana M., Singh M., Kumar P. (2016). Total antioxidant and oxidant status of plasma and renal tissue of cisplatin-induced nephrotoxic rats: Protection by floral extracts of *Calendula officinalis* Linn. Ren. Fail..

[391] Jędrejek D., Kontek B., Lis B., Stochmal A., Olas B. (2017). Evaluation of antioxidant activity of phenolic fractions from the leaves and petals of dandelion in human plasma treated with H_2_O_2_ and H_2_O_2_/Fe. Chem Biol Interact..

[392] Haghi G., Hatami A., Mehran M. (2013). UPLC and HPLC of caffeoyl esters in wild and cultivated *Arctium lappa* L.. Food Chem..

[393] Gentil M., Pereira J.V., Sousa Y.T., Pietro R., Neto M.D., Vansan L.P., de Castro França S. (2006). In vitro evaluation of the antibacterial activity of *Arctium lappa* as a phytotherapeutic agent used in intracanal dressings. Phytother. Res..

[394] Groppo F.C., Bergamaschi C.D.C., Cogo K., Franz-Montan M., Motta R.H., de Andrade E.D. (2008). Use of phytotherapy in dentistry. Phytother. Res..

[395] Tonea A., Badea M., Oana L., Sava S., Vodnar D. (2017). Antibacterial and antifungal activity of endodontic intracanal medications. Clujul Med..

[396] Jaimand K., Rezaee M.B., Mozaffarian V. (2006). Chemical constituents of the leaf and flower oils from *Achillea millefolium* ssp. *elbursensis* Hub.Mor. from Iran rich in chamazulene. J. Essent. Oil Res..

[397] Miranzadeh S., Adib-Hajbaghery M., Soleymanpoor L., Ehsani M. (2015). Effect of adding the herb *Achillea millefolium* on mouthwash on chemotherapy-induced oral mucositis in cancer patients: A double-blind randomized controlled trial. Eur. J. Oncol. Nurs. Off. J. Eur. Oncol. Nurs. Soc..

[398] Vitalini S., Beretta G., Iriti M., Orsenigo S., Basilico N., Dall’Acqua S., Iorizzi M., Fico G. (2011). Phenolic compounds from *Achillea Millefolium* L. and their bioactivity. Acta Biochim. Pol..

[399] Tyszkiewicz E.W. (2008). Assessment Report on Solidago virgaurea L., Herba; Evaluation of Medicines for Human Use.

[400] Laurençon L., Sarrazin E., Chevalier M., Prêcheur I., Herbette G., Fernandez X. (2013). Inhibition of *Candida albicans* yeast-hyphal conversion by triterpenoid saponins from the aerial parts of *Solidago virgaurea* alpestris. Phytochemistry.

[401] Prêcheur I., Rolland Y., Hasseine L., Orange F., Morisot A., Landreau A. (2020). *Solidago virgaurea* L. plant extract targeted against Candida albicans to reduce oral microbial biomass: A double-blind randomized trial on healthy adults. Antibiotics.

[402] Crestez A.M., Nechita A., Daineanu M.P., Busila C., Tatu A.L., Ionescu M.A., Martinez J.D., Debita M. (2024). Oral Cavity Microbiome Impact on Respiratory Infections Among Children. Pediatr. Health Med Ther..

